# Chemistry and Pharmacological Actions of Delphinidin, a Dietary Purple Pigment in Anthocyanidin and Anthocyanin Forms

**DOI:** 10.3389/fnut.2022.746881

**Published:** 2022-03-17

**Authors:** Asif Husain, Harshit Chanana, Shah Alam Khan, U. M. Dhanalekshmi, M. Ali, Anwar A. Alghamdi, Aftab Ahmad

**Affiliations:** ^1^Department of Pharmaceutical Chemistry, School of Pharmaceutical Education and Research, Jamia Hamdard, New Delhi, India; ^2^College of Pharmacy, National University of Science and Technology, Muscat, Oman; ^3^Department of Pharmacognosy, College of Pharmacy, Jazan University, Jizan, Saudi Arabia; ^4^Department of Health Information Technology, Faculty of Applied Studies, King Abdulaziz University, Jeddah, Saudi Arabia

**Keywords:** delphinidin, anthocyanidin, anthocyanin, health benefits, bioavailability

## Abstract

Anthocyanins are naturally occurring water-soluble flavonoids abundantly present in fruits and vegetables. They are polymethoxyderivatives of 2-phenyl-benzopyrylium or flavylium salts. Delphinidin (Dp) is a purple-colored plant pigment, which occurs in a variety of berries, eggplant, roselle, and wine. It is found in a variety of glycosidic forms ranging from glucoside to arabinoside. Dp is highly active in its aglycone form, but the presence of a sugar moiety is vital for its bioavailability. Several animal and human clinical studies have shown that it exerts beneficial effects on gut microbiota. Dp exhibits a variety of useful biological activities by distinct and complex mechanisms. This manuscript highlights the basic characteristics, chemistry, biosynthesis, stability profiling, chemical synthesis, physicochemical parameters along with various analytical methods developed for extraction, isolation and characterization, diverse biological activities and granted patents to this lead anthocyanin molecule, Dp. This review aims to open pathways for further exploration and research investigation on the true potential of the naturally occurring purple pigment (Dp) in its anthocyanidin and anthocyanin forms beyond nutrition.

## Graphical Abstract

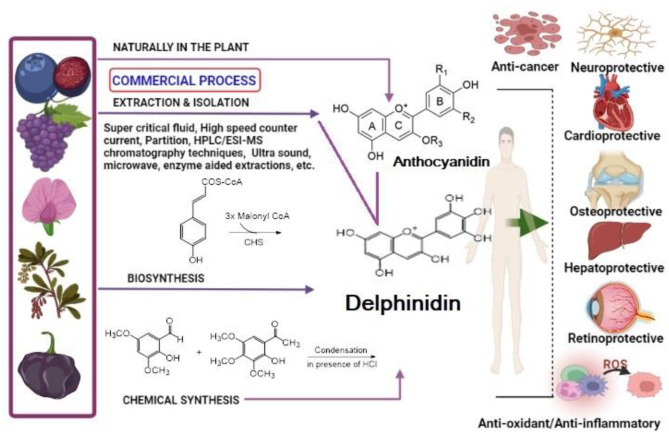

## Highlights

- It covers phytochemistry of delphinidin including biosynthesis, extraction, isolation, and analysis.
- Overview of biological activities of delphinidin and its glycosides with emphasis on molecular mechanism.
- It lists synergistic combinations of delphinidin with anticancer agents.
- It includes patents granted to delphinidin.

## Introduction

**Figure 1:**
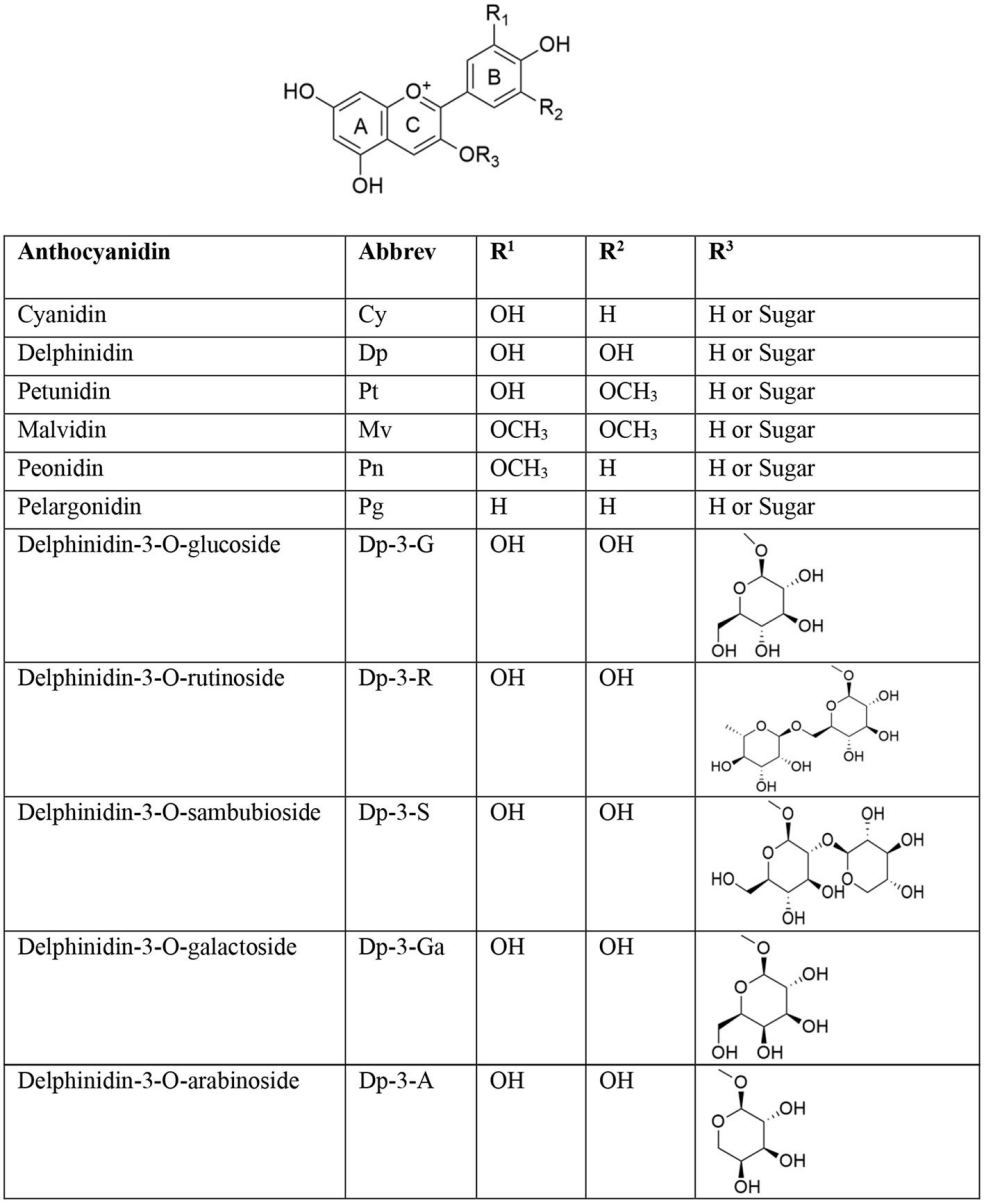
Major anthocyanins and glycosidic forms of delphinidin present in nature.

Flavonoids are one of the biggest classes of polyphenolic compounds having diverse chemical structures and characteristics that are present ubiquitously in plants. These plant pigments have a skeleton of 15 carbon atoms (C6-C3-C6) containing three rings, *viz*., 2 phenolic (A, B) and one pyran (C) rings (Figure 1) (1). The first classical report on isolation of blue anthocyanin from *Centaurea cyanus* (cornflower) was published in 1913 by Willstätter and Everest (2).

Anthocyanins (*anthos* means flower and *kyanos* means blue in Greek) belong to a class of water-soluble flavonoids and are natural pH indicators (3). They are commonly found in higher plants and are mainly accountable for the blue, purple, and red colors of fruits like berries, grapes, currants (gooseberries), some tropical fruits, vegetables, roots, and cereals. Acyl glycosides of anthocyanidin are biosynthesized *via* the phenylpropanoid pathway (4). Chemically, these compounds are polymethoxy or polyhydroxy derivatives of flavylium or 2-phenyl-benzopyrylium salts. Glycosidic moieties are present as mono-, di-, or triglycerides bonded by α or β glycosidic linkages. Glycosidic linkage is present in the C-3 position of the anthocyanidin (aglycone). Commonly present sugar moieties include glucose, galactose, rhamnose, arabinose, and xylose (5). The sugar part (glycone) of anthocyanins is responsible for chemical stability and solubility. Anthocyanin content in fruits and vegetables varies considerably and is found in the range of ~30–1,500 mg/100 g (6). An anthocyanidin is an aglycone moiety that is formed by hydrolysis of anthocyanin glycoside. Anthocyanins are ingested as components of complex mixtures of flavonoid components. Presence of flavylium ion and unusual electron distribution make anthocyanidins a highly unstable moiety; hence, the aglycone form of anthocyanins exists very rarely in nature (7).

Approximately, over 700 unique anthocyanins have been isolated so far (8). Most abundant anthocyanin aglycones (anthocyanidins) include peonidin (Pn), cyanidin (Cy), perlargonidin (Pg), malvidin (Mv), petunidin (Pt), and delphinidin (Dp), and they are of paramount importance. Anthocyanins are commonly used in food supplements and nutraceuticals because of their beneficial effects on humans. Anthocyanins exhibit a broad range of pharmacological activities, and they have antioxidant, anti-inflammatory (9), anticancer (10), anti-ulcer (11), cardioprotective (12), antidiabetic (13), and neuroprotective (14) properties. Anthocyanins are differentiated on the basis of number and nature of aliphatic or aromatic acids attached to sugars in a molecule, degree of methylation of hydroxyl groups, number of hydroxyl groups present, and nature, number, and location of sugars attached to a molecule (15). Conjugated double bonds present in an anthocyanidin moiety are responsible for absorption of light and production of unique colors. In general, methoxylation in an aromatic ring imparts a red color while increase in hydroxylation tends to make a pigment blue. In anthocyanidins, chief structural differences occur in the 3′ and 5′ positions of a B ring. Glycosides of Cy, Dp, and Pg are extensively distributed in nature. The distribution of Dp in edible plant parts is around 12% (16). Copious concentrations of Dp are present in blue and purple flowers, and Dp is biosynthesized along with Pg and Cy; principally, these are basic anthocyanidin skeletons of flower color pigments (17). Dp is a magenta- to purple-colored plant pigment and a dominant anthocyanidin found in blackcurrant, bilberry, blueberry, concord grape, eggplant, roselle, and wine (18). Chemical structures of some major and therapeutically important anthocyanidins are given in Figure 1 (7).

Dp (3,3',4',5,5',7-hexa-hydroxy-flavylium), one of the major anthocyanidins, is a polyphenolic compound with oxygen in the 1st position, and is linked to the sugar moiety in 3-O-β- position of the C ring. Dp (PubChem CID: 68245) is made up of three rings, *viz*, A (resorcinol), B (catechol), and C (3-O-subsituted-pyrylium). Dp has 6 hydrogen bond donor and 6 hydrogen bond acceptor atoms. Because of the presence of numerous electron donor atoms, Dp acts as a potent antioxidant by scavenging reactive oxygen species (ROS). The presence of a 3-hydroxyl group in ring B of Dp distinguishes it from other anthocyanins. It also possesses two hydroxy groups in ring A. These -OH groups are responsible for a variety of crucial biological activities, as they form potent interactions with a variety of proteins (19). Dp is more active in its aglycone form, but the presence of a sugar moiety in the 3rd position of the C ring is vital for its bioavailability (20). Dp is highly polar compared to most of anthocyanins owing to the presence of several hydroxyl groups and, thus, is easily soluble in methanol and water (21). Dp is linked to a variety of sugar moieties, ranging from glucoside to arabinoside in the C-3 position. Some major glycosidic forms of Dp present in nature are illustrated in Figure 1.

There has been a substantial increase in the frequency of publication of articles stipulating the therapeutic effectiveness of Dp and its glycosides over the years. This review article aimed to discuss the phytochemical aspects, biosynthesis, physiochemical characteristics, and potential therapeutic activities of Dp, and the progress made in research on this purple pigment and its glycosides.

## Phytochemical Aspects of Dp

### Biosynthesis

**Figure 2:**
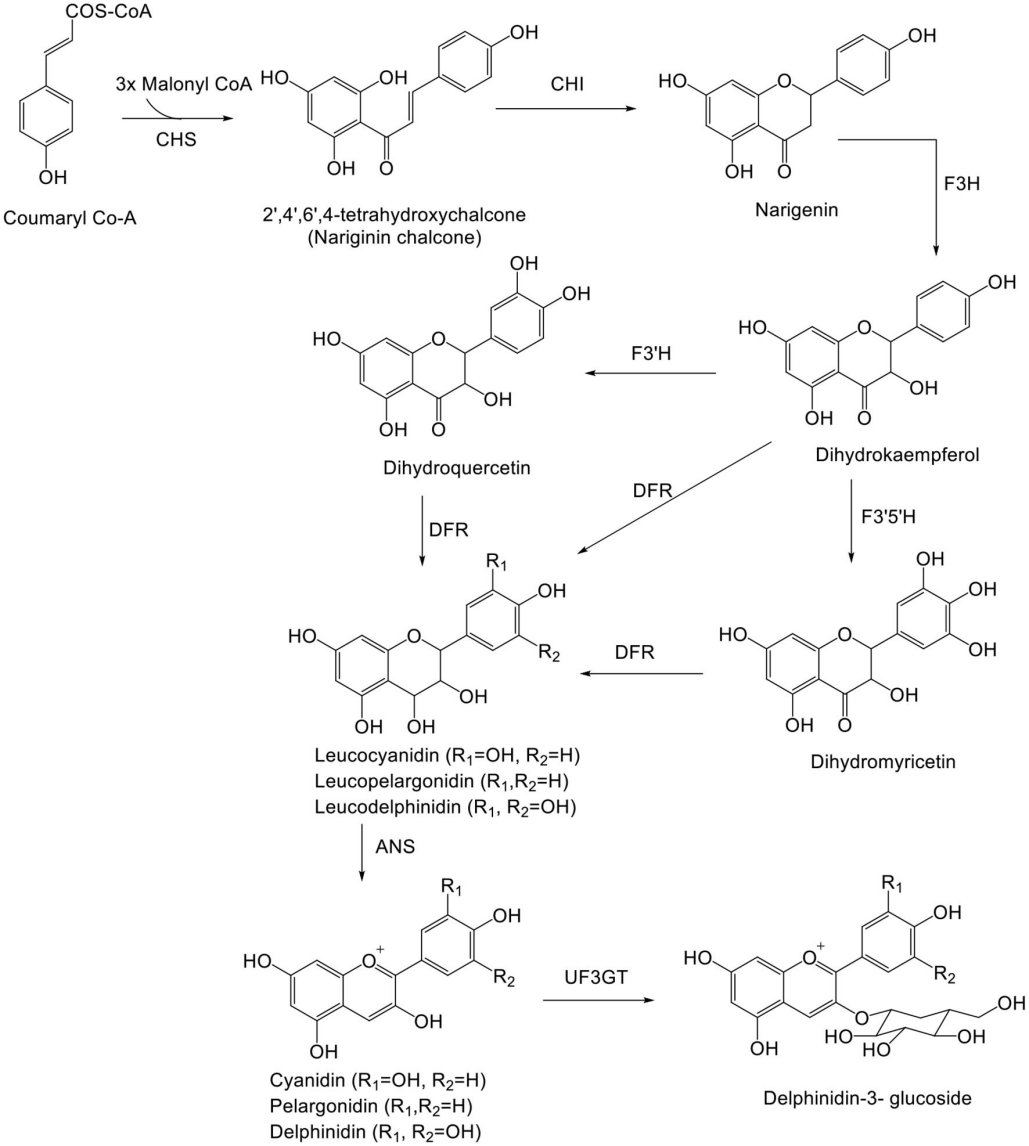
Biosynthetic pathway of delphinidin. ANS, anthocyanidin synthase; CHS, chalcone synthase; CHI, chalcone isomerase; F3H, flavanone 3-hydroxylase; F3′H, flavonoid-3′5′-hydroxylase; F3′5′H, flavonoid-3′,5′-hydroxylase; DFR, dihydroflavonol-4-reductase; UF3GT, UDP-Glc flavonoid 3-O-glucosyl transferase.

Delphinidin (Dp) is biosynthesized along with other anthocyanidins (Cy and Pg) from coumaroyl-CoA and malonyl-CoA (Figure 2), and 3',5'-hydroxylase is the key enzyme for Dp biosynthesis (22, 23). *De novo* assembly method used for biosynthesis of anthocyanin includes cinnamate-4-hydroxylase gene (C4H) and Chalcone synthase gene (CHS) and unigenes (24–26).

Delphinidin (Dp) is responsible for the magenta, purple, and blue colors of flowers. Dp, after biosynthesis, is glycosylated, acetylated, and methylated by glucosyltransferases, acyltransferases, and methyltransferases, respectively. Glycosylation of Dp takes place in the 3rd position. Methylation of Dp (anthocyanin) takes place in the 3' and 5' positions, resulting in formation of other anthocyanins (Pt and Mv) (27). UFGT and reductase enzymes compete to form anthocyanins and proanthocyanidins, respectively. Dp and leucodelphinidin undergo enzymatic reduction to epigallocatechin and gallocatechin, respectively. Following reduction, both epigallocatechin and gallocatechin or catechin (formed from leucocyanidin) undergo polymerization to form prodelphinidin. The enzyme responsible for polymerization is still unknown. Prodelphinidin is biosynthesized along with procyanidin (28).

Inspection of major metabolic pathways *via* chemical and transcriptomics analyses on Dp shows mutation of the ScbHLH17 and ScHI1/2 coding regions of anthocyanin formation in white yellow cultivars (29). Dp derivative-expressed ANS, F3'H, and DFR, genes have been determined by real-time quantitative (RT-q)PCR (30). Free anthocyanins in grapes are synthesized by the flavonoid pathway, which takes the similar upstream pathway with pro-anthocyanidins until formation of anthocyanins by catalysis of anthocyanidin synthase, also known as leucoanthocyanidin dioxygenase (7). On inspecting the mechanism of pre-harvest and post-harvest, UV showed pre-harvest UV-B, C and post-harvest UV-A, B, C irradiation lead to substantial anthocyanin biosynthesis in blueberry (31). Various metabolites identified by HPLC-MS specified that anthocyanin biosynthesis in purple-colored leaves was augmented, with maximum concentration of anthocyanidins, pro-anthocyanidins, and kaempferol glycoside (32). The biosynthetic pathway of Dp is outlined in Figure 2.

### Chemical Synthesis

**Figure 3:**
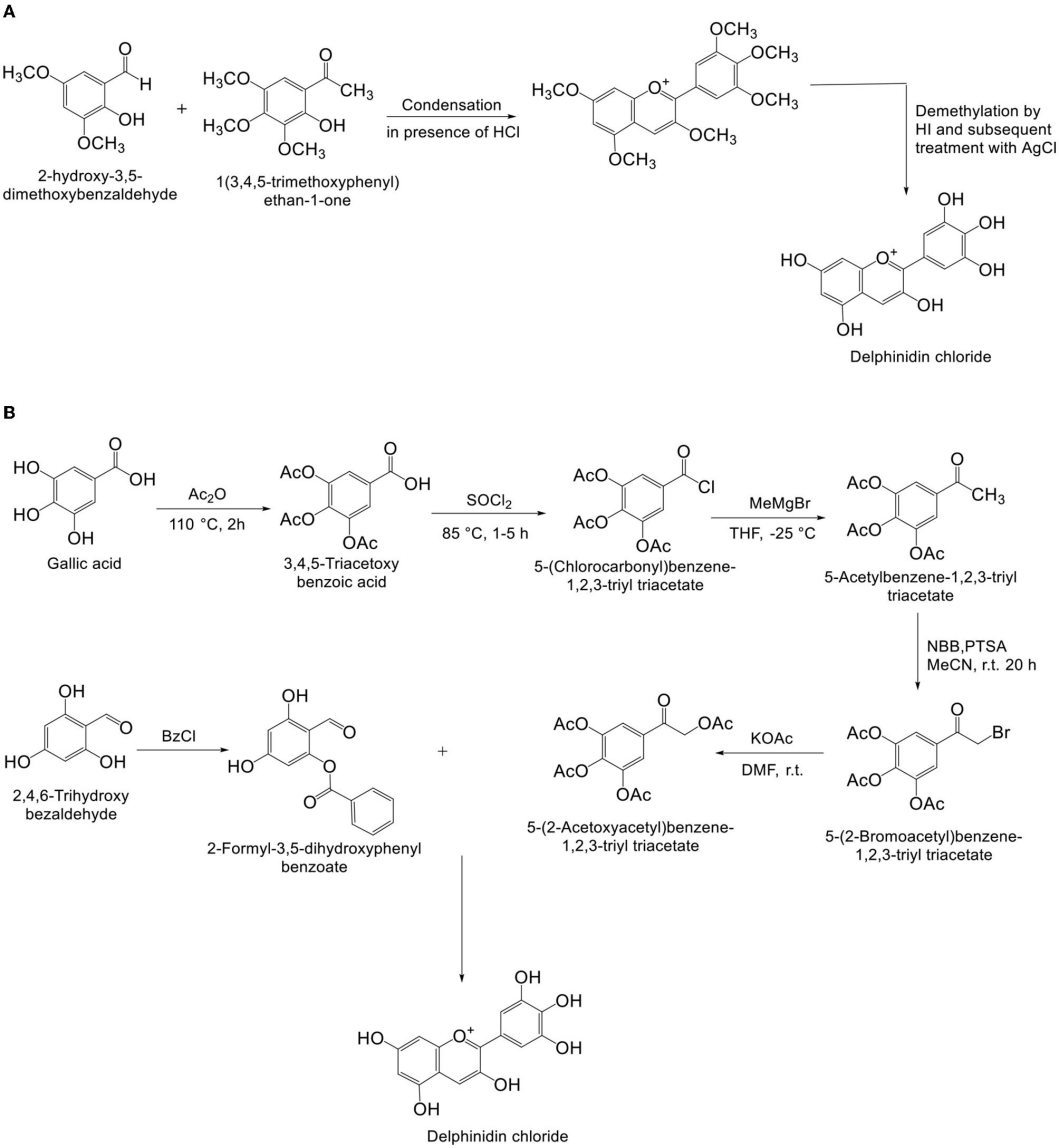
**(A,B)** Synthesis of delphinidin chloride.

Pratt and Robinson (33), first proposed the scheme for synthesis of various anthocyanins such as Dp (Figure 3A). Biosynth AS, on behalf of Bakstad et al. (34). filed a patent (US8513395B2) for anthocyanin synthesis in 2006, and the patent was granted in August 2013. Thiele et al. (35), proposed a method for synthesis of Dp (Figure 3B), which produced better yield and purity of Dp chloride than the scheme proposed by Kraus et al. (36). Synthesis of light-independent and light-inducible anthocyanins controlled by specified genes in grape was introduced by Ma et al. (37).

### Stability Profile

**Figure 4:**
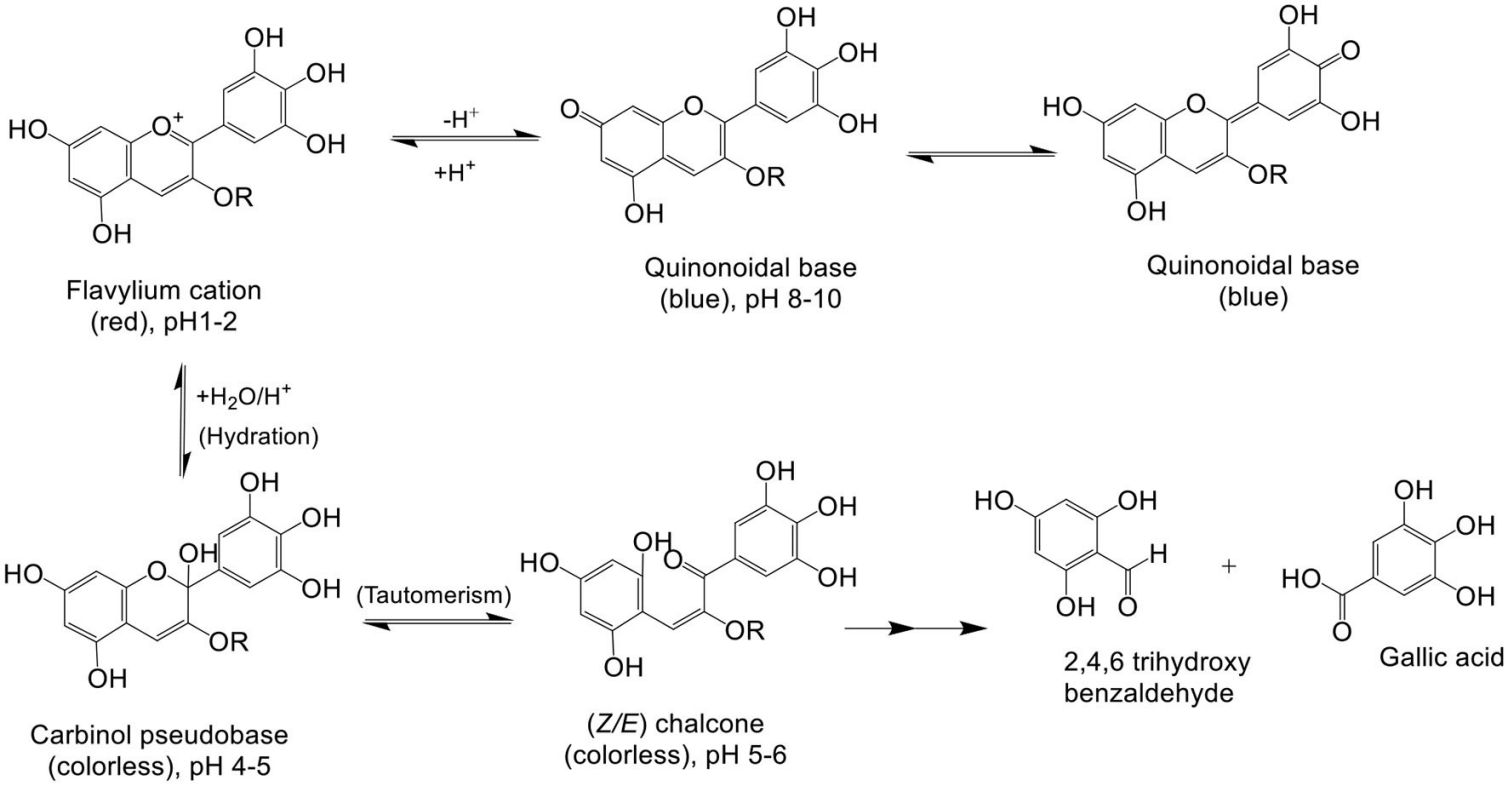
pH-mediated degradation of delphinidin: in low pH, 1–2 red colored flavylium cations are observed, in alkaline pH 8–10 blue-color quinonoidal bases, in pH 4–6 colorless carbinol pseudobase and chalcone, and in pH > 7, degradation products of delphinidin, 2,4,6-trihydroxybenzaldehyde and gallic acid, are observed (45, 46).

It is known that various chemical and environmental factors such as temperature, pH, light, and air affect the stability of anthocyanins, leading to easy degradation and decomposition during processing and storage (38). Anthocyanins degrade faster with increase in temperature. Dp, upon thermal degradation, undergoes B ring opening to produce an intermediate Dp chalcone by a first-order reaction. Dp chalcone further decomposes to produce 3,4,5 trihydroxybenzoic acid and phloroglucinaldehyde through B ring-retained and A ring-retained cleavages, respectively. Among various thermal degradation methods, HPLC-Q-TOF-MS analysis indicated that microwave causes highest rate of degradation as Dp content reduced from 100 to 43.2% in just 10 s followed by conventional heating and then ultrasound treatment. Trauner provided the first report regarding pH-dependent color change in anthocyanins (2). Dp is highly stable under acidic conditions but unstable in alkaline and neutral pH. Stability of Dp can be related to the presence of three-OH groups on ring B. The blue tinge in flowers is due to the presence of Dp pigment under alkaline conditions (39). Dp is a natural pH indicator, and it appears red in acidic pH, blue in basic pH, and purple to magenta in neutral pH (23). The color of Dp pigment is purple because substitution of the three hydroxyl groups on ring B that results in bathochromic shift of visible absorption maximum (λ_max_) to a longer wavelength (17). Nanogel encapsulation enhances the chemical stability of cyanidin-3-O-glucoside (Cy3G) by combining Maillard reaction and heat gelation (40, 41). Flavone co-pigments resulted in hyperchromic and bathochromic shifts, and a protective effect of flavone co-pigmentation was found in glycosides (42). Anthocyanins, cyanidin 3-*O*-β-rutinoside (Cy-3R), and cyanidin 3-*O*-β-glucoside displayed first-order degradation rates, presenting higher size of conjugated sugars (43). For interpretation of the absence of color phenotype in white-colored flowers of strawberry hybrids, a new hypothesis was pressed based on transcriptome analysis and the competitive effect of FpFLS and FpDFR genes that were shown to inhibit anthocyanin synthesis (44). The pH-mediated degradation pathway for Dp is illustrated in Figure 4.

### Physicochemical Aspects

Dp is highly soluble in polar solvents, and it increases with increase in temperature and polarity. The mole fraction solubility of Dp is highest in methanol (58.61 ± 0.01 to 168.64 ± 0.02) followed by water (53.53 ± 0.06 to 163.71 ± 0.02), ethanol (5.73 ± 0.02 to 15.59 ± 0.02), and acetone (0.0055 ± 0.0012 to 0.0157 ± 0.0013) at temperatures ranging from 298.15 to 343.15 K (21). Because of high polarity, Dp possesses low log_P_-values, which results in poor lipid solubility. Lipid solubility can be ameliorated by lipophilization of Dp. Márquez-Rodríguez et al. carried out lipophilization of Dp-3-S extracted from *Hibiscus sabdariffa* using octanoyl chloride. Three ester derivatives of Dp-3-S were prepared by subsequent esterification in the C-4' position of ring B of Dp moiety, C-4' and sambubioside moiety, and sambubioside moiety, respectively. Results evaluated by density functional theory demonstrated esterification of the sambubioside (sugar) moiety to yield the most fruitful results. It resulted in high solubility in lipophilic medium without interfering with physical properties of Dp, as lipophilization of the sugar moiety averts the loss of absorbance intensity and its excited states are same with its precursor, Dp-3-S (47). Glycosides of Dp showed poor stability profile compared to other anthocyanins (Cy, Pt, Pn, and Mv) because of the number of -OH groups in the B ring, whereas methoxy group substitution in ring B augmented the stability as in Pt, Pn, and Mv. Furthermore, Dp-3G was stable under gastric (acidic) conditions but presented poor stability under intestinal (basic) conditions (48).

### Extraction, Isolation, and Characterization

Earlier, anthocyanins were extracted with a traditional solvent-aided extraction method using alcohol and acid (4). Newer anthocyanin extraction techniques have been introduced like super critical fluid chromatography (SCFC) (49), ultrasound-aided extraction (50), microwave-aided extraction (51), accelerated solvent extraction (52), enzyme-aided extraction (53), and solid-phase extraction (54).

Dp is isolated and characterized by numerous chromatographic and spectroscopic techniques such as high-speed countercurrent chromatography (55), ionic liquid-modified countercurrent chromatography, partition chromatography (56), HPLC-ESI/MS (57), and NMR (50, 58). High-performance liquid chromatography-mass spectrometry (HPLC-MS) and liquid chromatography-tandem mass spectrometry (LC-MS/MS) are two of the most important hyphenated analytical methods practiced for qualitative and quantitative analyses of Dp. A general description of extraction, isolation, analysis, and characterization of Dp from various sources is presented in Table 1.

**Table 1:** Conditions required for extraction, isolation, and characterization of delphinidin and its glycosides.

**S. No**.	**Plant products containing Dp as the major anthocyanin**	**Method for extraction of anthocyanin**	**Analysis and characterization of anthocyanins**	**Concentration of Dp glycosides**	**References**
1	Highbush blueberry (*Vaccinium corymbosum* L.)	Solvent aided extraction using acetone and acetic acid (99:1 v/v) and further ultrasound assisted sonification for 15 min	**HPLC conditions column**: Zorbax SB-C18 column (50 × 4.6 mm, 5 μm) **Flow rate**: 0.4 mL/min **Mobile phase A**: 5% formic acid solution **Mobile phase B**: methanol **Injection volume**: 10 μL **DAD wavelength**: 190–600 nm **ESI-MS**	**Toro species** Dp-3-Ga: 7.68 ± 1.42 mg/100 g Dp-3-A: 1.63 ± 0.09 mg/100 g **Legacy species** Dp-3-Ga: 11.44 ± 3.70 mg/100 g Dp-3-A: 4.07 ± 1.15 mg/100 g **Duke species** Dp-3-Ga: 14.99 ± 3.97 mg/100 g Dp-3-A: 5.10 ± 1.22 mg/100 g **Bluecrop species** Dp-3-Ga: 2.29 ± 0.21 mg/100 g Dp-3-G: 1.21 ± 0.10 mg/100 g Dp-3-A: 1.66 ± 0.10 mg/100 g	(59)
2	Highbush blueberry (*Vaccinium corymbosum* L.)	Solvent assisted extraction with 87% v/v acetonitrile, 3% v/v water, 10% v/v formic acid from blueberry and its juice	**HPLC conditions column**: Phenomenex Luna C-18, 100A (250 × 4.60 mm, 3μ) **Column temperature**: 25°C **Flow rate**: 0.5 mL/min **Mobile phase A**: 87% v/v acetonitrile, 3% v/v water, 10% v/v formic acid **Mobile phase B**: 50% v/v acetonitrile, 40% v/v water, 10% v/v formic acid **DAD wavelength**: 520 nm	**Blueberry from Uruguay** Anthocyanins: 1,570 mg/100 g dry weight Dp-3-Ga: 298 mg/100 g Dp-3-G: 128 mg/100 g Dp-3-A: 142 mg/100 g **Blueberry from Poland** Anthocyanins: 2,242 mg/100 g Dp-3-Ga: 541 mg/100 g Dp-3-G: 11 mg/100 g Dp-3-A: 244 mg/100 g **Blueberry from Germany** Anthocyanins: 2,762 mg/100 g Dp-3-Ga: 543 mg/100 g Dp-3-G: 12 mg/100 g Dp-3-A: 250 mg/100 g	(60)
3	Lowbush blueberry (*Vaccinium angustifolium*)	Solvent assisted extraction carried using acidified ethanol and then further extracted by solid phase extraction carried out using Circa, C-18 adsorbent (modified silica gel distribution of particles 40–63 um, organic load 0.38 mmol g^−1^, carbon load 9.16%) dispersed in 95% ethanol	**Preparative HPLC conditions column**: XTerra Prep MS C-18 OBDTM (5 mm, 19 × 100 mm i.d., 5 μm) **Column temperature:** 25°C **Flow rate**: 2.0 mL/min **Mobile phase A**: 5% formic acid solution **Mobile phase B**: Methanol **DAD wavelength**: 520 nm	Anthocyanin: 485 mg of cyanin equivalent Dp-3-Ga: 16 mg of cyanin equivalent Dp-3-G: 31 mg of cyanin equivalent Dp-3-Ar: 19 mg of cyanin equivalent Dp-3-(p-coumaroyl)-G: 5 mg of cyanin equivalent Dp-3-(6″-acetyl)-G: 20 mg of cyanin equivalent	(61)
4	Lowbush blueberry (*Vaccinium angustifolium*)	Solid phase extraction using (SPE)-cartridge (Strata-X 300 mg/3 mL, Phenomenex) and elution was carried out using 0.01 N HCl (5 mL), ethyl acetate (10 mL), and acidified methanol (5 mL) with 0.1% HCl	**HPLC conditions column**: C-18 Kinetex column (150 × 4.6 mm, 2.6 μm) **Column temperature:** 45°C **Flow rate**: 1.7 mL/min **Mobile phase A**: 1 % H_3_PO_4_ **Mobile phase B**: acetonitrile/water (35:65 v/v) **DAD wavelength**: 200–700 nm, Anthocyanin (520 nm)	Anthocyanin: 29.9 ± 5.17 mg/mL Dp-3-G: 2.18 ± 0.7 mg/mL Dp-3-GA: 1.55 ± 0.15 mg/mL Dp-3-A: 0.75 ± 0.02 mg/mL	(62)
5	Bilberry (*Vaccinium myrtillus* L.)	Solvent aided extraction using ethyl acetate and maceration with acidified methanol (0.6 M HCl) to extract anthocyanin	**HPLC conditions column**: Zorbax SB-C-18 column (150 × 4.6 mm i.d., 5 um) **Column temperature**: 25°C **Flow rate**: 1.0 ml/min **Injection volume**: 5 ul	**Anthocyanins:** 568.8 ± 8.8 mg/100 g fresh weight Dp-3-Ga: 92.1 ± 4.7 mg/100 g Dp-3-G: 86.6 ± 10.5 mg/100 g Dp-3-A: 59.1 ± 4.8 mg/100 g	(63)
			**Mobile phase A**: water–formic acid (9:1 v/v) **Mobile phase B**: methanol-water-formic acid(5:4:1 v/v/v) **DAD wavelength**: 190–650 nm **ESI+/MS & MS**^**2**^ **conditions**		
6	Bilberry (*Vaccinium myrtillus* L.)	Solvent assisted extraction with 87% v/v acetonitrile, 3% v/v water, 10% v/v formic acid from blueberry and its juice	**HPLC conditions column**: Phenomenex Luna C-18, 100A (250 × 4.60 mm, 3 μm) **Column temperature**: 25°C **Flow rate**: 0.5 mL/min **Mobile phase A**: 87% v/v acetonitrile, 3% v/v water, 10% v/v formic acid **Mobile phase B**: 50% v/v acetonitrile, 40% v/v water, 10% v/v formic acid **DAD wavelength**: 520 nm	**Bilberry from Poland (sample I)** Anthocyanin: 6,102 mg/100g dry weight Dp-3-Ga: 847 mg/100 g Dp-3-G: 1,047 mg/100 g Dp-3-A: 603 mg/100 g **Bilberry from Poland (sample II)** Anthocyanin: 7,465 mg/100 g Dp-3-Ga: 1,060 mg/100 g Dp-3-G: 1,247 mg/100 g Dp-3-A: 741 mg/100 g	(60)
7	Bilberry (*Vaccinium myrtillus* L.)	Extraction carried out using 60% ethanol acidified with HCl Purification by macroporous adsorbent (Amberlite XAD-7, XAD-4, AB-8, ADS-17, and DE- 5-40)	**HPLC conditions column**: Venusil ASB C18 (250 × 4.6 mm, 5 μm) **Column temperature**: 30°C **Flow rate**: 1 mL/min **Mobile phase A**: anhydrous formic acid/water (8.5:91.5 v/v) **Mobile phase B**: anhydrous formic acid/acetonitrile/methanol/water (8.5:22.5:22.5:41.5 v/v/v/v) **DAD wavelength**: 535 nm	ND	(64)
8	Blackcurrant (*Ribes nigrum* L.)	Solvent aided extraction using acetone and acetic acid (99:1 v/v) and further ultrasound assisted sonification for 15 min	**HPLC conditions column**: Zorbax SB-C18 column (50 × 4.6 mm, 5 μm) **Flow rate**: 0.4 mL/min **Mobile phase A**: 5% formic acid solution **Mobile phase B**: methanol **Injection volume**: 10 μL **Gradient conditions**: 5% B (5 min), 5–50% B (45 min), 50–75% B (55 min), 75–100% B (65 min) **DAD wavelength**: 190–600 nm **ESI+/-/MS**^**3**^ **conditions capillary voltage:** 3,100 V **Capillary temperature**: 325°C **Nebulizing gas** (**N2) pressure**: 50 psi **Nebulizing gas (N2) flow rate**: 12 L/min **Collision gas (He) voltage ramping cycle**: 0.3–2 V	**Rosenthal species** Anthocyanin: 180.44 ± 3.59 mg/100g Dp-3-G: 16.86 ± 4.07 mg/100g Dp-3-R: 89.66 ± 8.33 mg/100g **Rovada species** Anthocyanin: 162.83 ± 2.46 Dp-3-G: 13.92 ± 7.33 mg/100 g Dp-3-R: 65.27 ± 25.39 mg/100 g	(59)
9	Blackcurrant (*Ribes nigrum* L.) pomace	Extraction with methanol:water:formic acid (50:48:2 v/v/v)	**HPLC conditions column**: Gemini 5u C-18 110A column (150 × 4.6 mm i.d., 5 μm) **Column temperature**: 40°C **Flow rate**: 1 mL/min **Injection volume**: 20 μl **Mobile phase A**: 10% v/v formic acid solution **Mobile phase B**: acetonitrile:water:formic acid (50:40:10 v/v/v) **DAD wavelength**: 520 nm	**Anthocyanin content from two harvest seasons (2006–2007) fraction 0.8** Anthocyanin-344.6 ± 0.5–729.7 ± 4.4 mg/100 g Dp-3-G: 85.3 ± 1.2–190.9 ± 0.3 mg/100 g Dp-3-R: 122.2 ± 1.7–254.2 ± 3.4 mg/100 g **Fraction 2–5** Anthocyanin: 577.1 ± 32.2–1,046.1 ± 10.4 mg/100 g Dp-3-G: 150.7 ± 8.6–288.5 ± 8.1 mg/100 g	(65)
				Dp-3-R: 213.9 ± 12.9–369.3 ± 2.6 mg/100 g **Fraction 5** Anthocyanin: 513.7 ± 26.4–911.7 ± 13.1 mg/100g Dp-3-G: 133.1 ± 5.9–249.9 ± 7.6 Dp-3-R: 191.4 ± 13.2–320.2 ± 1.3	
10	Blackcurrant (*Ribes nigrum* L.) pomace	Extraction with acidified water (0.01% v/v concentrated HCl) and purification with Amberlite XAD-7HP resin and elution with acidified ethanol (0.01% v/v conc. HCl). Further extraction with acidified water, isopropylacetate and ethyl acetate	**HPLC conditions column**: Zorbax Eclipse XDB C18 (150 × 4.6 mm, 5 μm) **Flow rate**: 1 mL/min **Injection volume**: 10 μL **Mobile phase A**: water (0.5% TFA) **Mobile phase B**: acetonitrile (0.5% TFA) **DAD wavelength**: 190–600 nm and for anthocyanin-520 nm **Preparative HPLC conditions column**: XBridge Prep C18 (10 × 50, 5 μm) **Flow rate**: 5 mL/min **Injection volume**: 300 μL Rest conditions were similar to analytical HPLC	Concentration of Dp-3-R (22.6%) was highest followed by Cy-3-R (20.4%), Dp-3-G (7.7%), Cy-3-G (4%)	(66)
11	Eggplant (*Solanum melongena* L)	Sadilova et al. reported the extraction from eggplant peel with aqueous acetone maintained at pH 1 i.e., water acidified with trifluoroacetic acid/acetone, 30:70, v/v Solvent assisted extraction employing methanol with 3% trifluoroacetic acid Dranca et al. reported the ultrasound assisted extraction from eggplant peel with frequency of 12.5, 25, and 37.5 KHz at temperature of 50, 60 and 70°C with extraction time of 15, 30, and 45 min and employing solvents methanol and 2-propanol	**HPLC conditions column**: C18 sunfire column, Waters (250 × 4.6 mm, 5 μm) and a C18 pre-column, Phenomenex (4 × 3.0 mm i.d.) **Column temperature**: 25°C **Flow rate**: 1 mL/min **Mobile phase A**: 5% formic acid solution **Mobile phase B**: 100% acetonitrile **DAD wavelength**: 520 nm	Dp-3-R: 378 ± 9.9 mg/kg Dp-3-G: 16.5 ± 1.0 mg/kg Dp-3-R-5G: 36.5 ± 0.8 mg/kg Dp-3-R-G: 19.1 ± 0.1 mg/kg.	(67, 68)
12	Violet pepper (*Capsicum annuum* L.) peel	Extraction from voilet pepper peel with aqueous acetone maintained at pH 1 i.e., water acidified with trifluoroacetic acid/acetone, 30: 70, v/v Solvent assisted extraction employing methanol with 3% trifluoroacetic acid	**HPLC conditions column**: C18 sunfire column, Waters (250 × 4.6 mm, 5 μm) and a C18 pre-column, Phenomenex (4 × 3.0 mm i.d.) **Column temperature**: 25°C **Flow rate**: 1 mL/min **Mobile phase A**: 5% formic acid solution **Mobile phase B**: 100% acetonitrile **DAD wavelength**: 520 nm Characterization was further done by ESI-MS	Dp-3-R-5-G: 36.5 ± 0.8 mg/kg Dp-3-G: 2.2 ± 0.0 mg/kg Dp-3-R: 2.9 ± 0.2 mg/kg Dp-3-rhamnoside: 2.4 ± 0.5 mg/kg Dp-3-caffeoyl-rutinoside-5-G: 7.8 ± 0.4 mg/kg Dp-3-cis-coumaroyl-rutinoside-5-G: 14.7 ± 0.8 mg/kg Dp-3-trans-coumaroyl-rutinoside-5-G: 286.2 ± 1.7 mg/kg Dp-3-feruloyl-rutinoside-5-hexose: 3.8 ± 0.1 mg/kg	(67)
13	*Hibiscus sabdariffa* (Roselle)	Solvent-aided extraction from *Hibiscus sabdariffa* dried calyces using distilled water as a solvent	**HPLC conditions column**: Phenomenex Gemini C18 column (250 × 4.6 mm, 5 μm) **Column temperature**: 35°C **Flow rate**: 1 mL/min **Injection volume**: 10 μl **Mobile phase A**: 0.1% (v/v) trifluoroacetic acid **Mobile phase B**: trifluoroacetic acid/acetonitrile/water (50:49.9:0.1) **DAD wavelength**: 265–520 nm	**Dark red variety** Anthocyanin: 2,732 ± 260 mg/100 g Dp-3-S: 2,116 ± 216 mg/100 g Dp-3-G: 76 ± 8 mg/100 g **Light red variety** Anthocyanin: 727 ± 55 mg/100 g Dp-3-S: 535 ± 37 mg/100 g Dp-3-G: 38 ± 1 mg/100 g	(18)

### Pharmacokinetic (Absorption, Distribution, Metabolism, and Excretion) Profile of Dp

Absorption and metabolism of anthocyanins rely on aglycones and type of sugar moiety present (69). Bioavailability of Dp is mainly governed by the type of sugar moiety present and its metabolism. A galactoside moiety possessed the highest bioavailability followed by glucoside and arabinoside (70). Dp-3R and Dp-3G are absorbed, distributed, and excreted in intact form along with the sugar moiety. Minor amounts of the metabolite 4′-O-methyl-Dp-3R were also excreted in urine. Bioavailability of orally administered Dp-3R is very poor, i.e., 0.49 ± 0.06% and 0.5% for oral and IV administration, respectively. Plasma C_max_ value is maximum for anthocyanins with rutinoside moiety (71–73). Dp-3G is absorbed in intact form and appears in the blood plasma 15 min after oral administration, and another absorption peak is seen after 60 min. Dp-3G is metabolized by methylation of the 4′ OH group in B-ring by COMT, and the metabolite exhibits better distribution profile (74). Dp-3G gets absorbed in the stomach and upper part of the small intestine because of bacteria and enzymes in the small intestine and partly because of early methylation of the glucoside (75). Intake of carbohydrate rich diet, delayed absorption and excretion of Dp-3R and Dp-3G in blackcurrant is due to longer transit time in GIT (76). Gallic acid is expected to be a major product of Dp-3G metabolism, but the literature by Nurmi et al. revealed that no gallic acid metabolite was observed in human urine although other metabolites were detected (77). Another study illustrated that Dp is unstable in its aglycone form, and that it has a half-life of <30 min. LC-MS/MS data manifested that Dp (aglycone), within 30 min, was rapidly converted into gallic acid and 2,4,6-trihydroxybenzaldehyde (78). The Dp present in blueberry and Kenyan purple tea crossed the blood-brain barrier and reached the central nervous system (CNS) in its intact glycosidic form. Availability of Dp was low in the CNS compared to other anthocyanins because of lower log_*P*_-value (79, 80). A bioavailability study conducted on Delphinol^®^ (powder extract of maqui berry) showed that Dp-3G could only reach a concentration of 0.64 μg/ml in the systemic circulation of human healthy volunteers, and that its maximum concentration was observed at 1 ± 0.3 h. C_max_ values ranged from 21.39 to 63.55 nmol/L (81).

Dp glycosides such as Dp-3G, Dp-3R, Dp-3-Gal, and Dp-3-Ar, are comparatively more bioavailable than Dp, which suffers from low bioavailability owing to its poor aqueous solubility (82). Bioavailability of Dp glycosides was found in the following order: Dp-3-Gal (0.48%) > Dp-3G (0.14%) ~ Dp-3-Ar (0.14%).

The low bioavailability, instability in physiologic pH, and high reactivity of Dp pose major challenges to the scientific community. Various approaches have been tried to improve the bioavailability of Dp so this naturally occurring plant pigment can find applications as a dietary supplement and nutraceutical in the food and pharmaceutical industries. Attempts have been made to improve its solubility, stability, and bioavailability by complexation with cyclodextrins such as sulfobutylether-β-cyclodextrin (83), microencapsulation with natural polymers, multiple emulsions, and nanoformulations (84–86). Dp nanoliposomes prepared by mingling cholesterol with a dried lipid layer of soy lecithin was better in reducing albumin glycation (91.5%) than Dp (69.5%) (87).

### Metabolic Characteristics of Dp and Its Effect on Gut Microbiota

Anthocyanins and anthocyanidins are reported to be absorbed from the intestine after ingestion; they interact with the transport proteins OATP1B1 and OATP1B3 and get transported to the liver for metabolism and excreted in the bile (88). Anthocyanins undergo metabolic biotransformation with the help of catechol-O-methyl transferase (COMT) in the gut and liver. Gut microbiota are mainly responsible for the biotransformation of most dietary anthocyanins that are not absorbed in the upper gastrointestinal tract. *Lactobacillus* bacteria are primarily involved in the metabolism of Dp in the colon. They use intestinal gut enzymes, like glucosidase, glucuronidase, galactosidase, and rhamnosidase, to cleave the glycosidic bond of Dp glycosides (anthocyanin) and to set the aglycone (anthocyanidin) part free. Gut microbiota allow the absorption of Dp and other flavonoids, and enhance their bioavailability (89). Pharmacokinetic information on Dp indicated that it reaches peak plasma value after 2 h of ingestion, and that the phase II metabolite of Dp glucuronide reaches peak level after 6.3 h (14). In a tissue culture medium, Dp degrades rapidly with a half-life of ~30 min into gallic acid and phloroglucinol aldehyde. Human primary hepatocytes and liver microsomal enzymes like CYP2C6, CYP2A6, CYP2B6, and CYP3A4 have been used for evaluation of metabolic characteristics of anthocyanins and anthocyanidins (88). It has been noted that Dp at a concentration of 100 μM significantly inhibits 90% of CYP3A4 catalytic activity. Glucosides of Dp inhibit the activity of CYP2C9 by 55%, and Dp-3-rut reduces the activity of CYP3A4 by 35% (90). Dp also inhibits glutathione S transferases (GSTs), carbonyl reductases (CBRs), and UDP-glucuronsyl transferases (UGTs).

Dp was found to possess significant noncompetitive inhibitory effects against human CBRs with an IC_50_ value of 16 μM and a substrate concentration of 500 μM, and to moderately and mildly inhibit UGTs and GSTs (IC_50_ = 150 μM), respectively. These inhibitory potencies are significantly different in rat and humans' samples. Differences in the metabolic pathway of Dp and its glucosides have also been reported. Dp 3, 5 di-glucoside can reduce the expression of SLCO/OATP1B3, but this effect has not been reported for Dp, indicating a correlation between the presence and the absence of a sugar moiety. Some factors like oxygen, polyphenols, and metals influence the metabolic degradation pathway of Dp analogs (14).

Consumption of high-fat diet, fruits, and vegetables rich in bioactive phenolic compounds are known to affect the gut microbiome, and significant changes in the population of gut microbiota could either increase or decrease the risk of chronic diseases. The interaction of gut microbiota with anthocyanins plays a prominent role in regulating homeostasis and the prebiotic activity of gut microbiome and its composition and population. One study reported that consumption of berries containing high content of Dp promoted proliferation of an oxygen-sensitive bacterial population by decreasing oxygen tension in gut lumens of mice (91). Igwe et al. carried out a systematic literature review to study the effect of anthocyanins on a population of gut mircobiota by including three *in vitro*, two *in vivo* animal model studies and one human interventional study. They concluded that anthocyanins exert beneficial effects on the population of gut microbiota, especially on proliferation of *Bifidobacterium spp*. and *lactobacillus-Enterococcus spp*., and on inhibition of a species of pathogenic bacteria, *Clostridium histolyticum*. *Bifidobacterium spp*. and *lactobacillus spp*. are widely used in probiotics to treat irritable bowel syndrome (IBS), enterocolitis, and diarrhea, and to exert beneficial effects on colorectal cancer (92). Dp-3G has been shown to significantly inhibit the population of the *C. histolycum* group (93). However, only a limited number of *in vitro, in vivo*, and human studies have been conducted so far; thus, it cannot be generalized that consumption of fruits and vegetables rich in Dp or supplementation with Dp leads to a favorable effect. More detailed animal and human studies are needed to confirm the beneficial effects of consumption of anthocyanin-/anthocyanidin-rich food including Dp on proliferation of healthy anaerobic gut microbiota. Also, the interaction between simultaneously administered drugs and high dose of anthocyanidin dietary supplement should be studied in greater detail to decide for a pharmacotherapeutic treatment plan. Although many reports defined potential interactions between drugs and anthocyanin supplements, their deep molecular metabolic reactions and safety concerns are not well-described; hence further research is warranted in the future.

## Therapeutic Potential and Health Benefits of Dp

### Anticancer Activity

Dp as both anthocyanidin and anthocyanin exhibits dominant anticancer activity against a variety of cancers such as breast, ovarian, colon, prostate, lung, hepatic, bone, blood, and skin cancers. Data from various literatures highlighting the anticancer potential of Dp and some of its glycosides are presented below. In majority of cancers, Dp acts by interfering with protein targets of the PI3K/Akt/mTOR and MAPK signaling pathways. The plausible mechanism of action of anticancer activity is illustrated in Supplementary Figures 1, 2).

#### Dp in Breast Cancer

Hepatocyte growth factor (HGF) and Met are potential candidate targets for therapeutic and pharmacological intervention in breast cancer therapy. Pretreatment of the human mammary epithelial cell line (MCF-10A) with Dp (5–40 μM) for 3 h inhibited the stimulation of Met expression. An immunoblot analysis revealed that Dp treatment down-regulated the expression of downstream proteins involved in regulating cell viability stimulated by HGF/Mets like FAK and Src, paxillin, CrkII and CrkL, Gab1, and SHP-2. Dp also prevented phosphorylation of Raf-1, MEK1/2, ERK1/2, STAT3, AKT, mTOR, p70S6K, and eIF4E, and the expression of PI3K85 stimulated by HGF. Consequently, Dp prevented HGF-provoked NF-κB and PKCα signaling (94).

Ozbay and Nahta observed that Dp (12.5, 25, 50, and 100 μg/ml) could induce apoptosis in seven breast cancer cell lines including ER negative (HCC1806, MDA231, MDA468, SKBR3, and MDA453) and ER positive (BT474, MCF7, MCF10A), but that apoptosis was not induced in non-transformed MCF10A cell lines. Rate of apoptosis was noted to be highest in human epidermal growth factor receptor-2 (HER2)-over expressing cell lines SKBR3 and BT474. Dp also prevented anchorage independent growth, proliferation, and metastasis, and further impeded HER2 and extracellular signal-regulated kinases ERK1/2 signaling in triple negative (MDA231 and MDA468) and HER2-overexpressing cell lines. Growth and ERK-1/2 signaling in transformed MCF10A cells were inhibited by Dp at doses of 25 and 50 μg/ml. Combination of Dp with approved drugs targeting HER-2 (Herceptin and lapatinib) was not effective compared to Dp treatment alone on SKBR3 and BT474 (95).

Chen et al. revealed an immense potential of Dp in the treatment of HER2-positive breast cancer cells. Dp was noted to be active against MDA-MB-453 and BT474 cells (IC_50_ = 40 and 100 μM, respectively), and induced caspase-9 and caspase-3 mediated apoptosis. TEM data showed that increase in Dp concentration reversed 3-methyladenine and Bafilomycin A1-provoked autophagic suppression and enhanced the expression of autophagic proteins (LC3-II, Atg5-Atg12 conjugate). Dp restrained the phosphorylation of p70S6K and eIF4E (proteins involved in mTOR signaling pathway) and played a role in the activation of ULK1 and FOXO3a (involved in LKB1-AMPK signaling pathway) (96).

Matrix metalloproteinase (MMP)-9 is responsible for metastasis of cancer cells. Dp at a concentration of 60 μM could inhibit MMP-9 expression stimulated by phorbol-12-myristate 13-acetate (PMA) in human breast carcinoma cells (MCF-7). Dp treatment inhibited proteins (p38 and JNK) involved in MAPK signaling pathway; it hindered the PMA-induced expression of c-Jun (AP-1 subunit) and p65 (NF-κB subunit), and IκBα degradation, leading to deactivation of nuclear factor-κB (NF-κB) and AP-1 (97).

Interestingly, Dp-3G treatment did not induce cytotoxicity in MCF10A and a human vascular endothelial cell line (EA. hy926), but cytotoxicity was induced to some extent in breast cancer cell lines like MCF-7, MDA-MB-453, and MDA-MB-231 by Dp-3G (0–40 μM). The anticancer effects of Dp-3-G were due to suppression of Akt stimulation and enhanced IRF1 expression which are involved in controlling HOTAIR expression in carcinogen treated MCF10A, other breast cancer cells and in xenografted breast tumor. A ChIP-qPCR analysis showed that treatment with Dp-3G enhanced the binding of IRF1 with HOTAIR promoter in MDA-MB-231 cells. Suppression of IRF1 expression by IRF1 siRNAs (TCanti-IRF1, stimulated HOTAIR expression and reduced the anticancer effects of Dp, signifying that Dp interferes with the regulation of Akt/IRF1/HOTAIR signaling pathway in breast cancer (98).

Han et al. investigated the anticancer potential of orally administered Dp (100 mg/kg/day) in female Sprague-Dawley rats with 1-methyl-1-nitrosourea (MNU)-induced breast cancer. Dp effectively reduced cancer incidence in the experimental animals by 43.7% and significantly decreased the rate of *in vitro* cell proliferation in MDA-MB-231, MCF-7, and MDA-MB-453 cell lines. The expression of Ki-67 (a nuclear protein that indicates cellular proliferation) and HOTAIR was substantially high in a control group in comparison with a group administered with Dp. Downregulation of HOTAIR and EZH2 and H3K27me3 expression with Dp resulted in upregulation of miR-34a in breast cancer cells and MNU-treated rats (99).

#### Dp in Ovarian Cancer

Lim et al. studied the role of Dp in ovarian cancer. The results of terminal de-oxynucleotidyl transferase dUTP nick end labeling (TUNEL) assay indicated that treatment with Dp (10 μM) could effectively suppress growth and migration, and that it was able to induce apoptosis in ovarian cancer cell line (ES2). Downstream proteins of the PI3K/AKT and p38 MAPK signaling pathways like AKT, ERK1/2, and JNK were effectively down-regulated, and the expression of proteins like p38 and GSK3b was not affected by Dp treatment (100). They observed synergism between a Dp-PI3K inhibitor (LY294002) and a Dp-p38 MAPK inhibitor (SB203580) against ES2 cells, whereas combination with an ERK1/2 MAPK inhibitor (U0126) was not significant (101). Subsequently, in 2017, their group demonstrated a cytotoxic effect of Dp (10 μM) on ovarian adenocarcinoma cell line (SKOV3), and downregulation of the expression of AKT, p70S6K, and S6 involved in PI3K pathway and proteins involved in MAPK/ERK1/2 and p38 MAPK pathway. It was noted that Dp treatment did not affect JNK expression, but that it enhanced the number of SKOV3 cells in sub-G1 phase and reduced cells in G_0_/G_1_ and G_2_/M phases. Synergism was observed between Dp-UO126 and Dp-LY294002, as the combination averted the proliferation and expression of ERK1/2 and p70S6K in SKOV3 cells more effectively. Combination of Dp-UO126 caused a tremendous increase in necrotic cells by ~886% and increased the number of SKOV3 cells in sub-G1 phase. Dp-LY294002 exhibited enhanced rate of apoptosis, increased the cells in S and G_2_/M phases compared to Dp alone, whereas combination of Dp-SB203580 was not much effective. These results indicate that Dp acts by inhibiting PI3K/AKT and ERK1/2 MAPK signaling in SKOV3 cells, and that their inhibition leads to both apoptosis and necrosis. The combination of Dp (10 μM) and paclitaxel (20 μM) was proved to be effective against SKOV3 cells compared to paclitaxel alone (102).

Brain-derived neurotrophic factor (BDNF)-treated SKOV3 cells (100 nM for 24 h) led to increased rate of cell growth and metastasis. Dp, at higher doses of 100 and 200 μM for 24 h, averted BDNF-provoked cell growth, and at a dose of 50 and 75 μM prevented the mobility and invasion of SKOV3 cells. Dp also suppressed the expression of BDNF-provoked metastasis-inducing proteins such as MMP9 and MMP2 (102).

#### Dp in Colorectal Cancer

Treatment of human colon cancer (HCT116) cells with Dp (30–240 μM, 48 h) decreased growth and proliferation, and enhanced apoptosis in a dose-dependent manner (IC_50_ 110 μM). Dp treatment not only cleaved poly(ADP-ribose) polymerase (PARP), it also attenuated the expression of procaspases–3, –8, and –9. It was observed to interfere with proteins involved in modulation of apoptosis, i.e., led to downregulation of Bcl-2 and upregulation of Bax expression. The number of cells in the G_2_/M phase of cell cycle was increased upon increasing the dose of Dp. Cell cycle arrest in the G_2_/M phase was due to reduction in expression of cyclin B1 and cycle 2 kinase and enhanced expression of p53 and p21WAF1/Cip1. NF-κB activation was hindered by Dp treatment of HCT116 cells (103).

Aichinger et al. reported that Alternaria mycotoxin alternariol (AOH), Dp, and genistein exhibit cytotoxic effects on HT-29 cells at 25, ≥25, and ≥ 25 μM, respectively. Synergism was observed between AOH and Dp or genistien, as Dp and AOH in combination exhibited greater toxicity toward HT-29 cells (104).

Altertoxin II (ATX-II) is a genotoxic impurity found in a variety of food products and is mainly responsible for DNA damage in colon carcinoma cells. ATX-II-Dp combination at doses ≥ 5 μM exhibited cytotoxicity in colon carcinoma cell line (HT-29). ATX-II (1 μM, 1 h)-provoked genotoxicity in HT-29 cells was successfully averted by Dp treatment at 50 and 100 μM, both in the presence and the absence of formamidopyrimidin-DNA-glycosylase. Dp (1 μM) also relieved oxidative stress because of generation of ROS by ATX-II (10 μM) in HT-29 cells. An LC-MS analysis revealed that Dp, gallic acid, and phloroglucinol aldehyde led to reduction in levels of ATX-II in solvents like PBS and DMEM but not in DMSO, signifying that Dp undergoes pH-mediated degradation (105).

Huang et al. (106) reported that Dp (50 and 100 μM) dose-dependently can reduce the number of colonies that form in soft agar by three cell lines, DLD-1, SW480, and SW620. Dp (100 μM) further reduced the number of attached SW620 cells, inhibited metastasis in DLD-1 and SW480 cells, downregulated the expression of MMP-2, and upregulated the expression of E-cadherin. In DLD-1 cells, Dp inhibited mir-204-3p expression by suppressing the expression of integrins αV and β3, which in turn suppressed FAK phosphorylation (Tyr397), Src phosphorylation (Tyr416), paxillin phosphorylation (Tyr31, Tyr118, and Tyr181), tensin and talin (integrin-associated adaptor proteins), Rac-1, Cdc42, and Rho A. *In vivo* studies on male Balb/c nude mice confirmed that Dp (100 μM) prevented metastasis in DLD-1 cells (106).

Recently, Zhang et al. reported that antiproliferative effects of Dp on colon cancer cells (HCT116) are due to apoptosis induction by modulation of the JAK/STAT3 and MAPKinase signaling pathways. Dp treatment is also associated with induction of cytochrome C, Caspase- 3, 8, and 9, and pro-apoptotic Bax, and it was found to inhibit anti-apoptotic protein expression (107).

#### Dp in Prostate Cancer

Jeong et al. (108) evaluated the cytotoxic effects of Dp against various prostate carcinoma cell lines. TUNNEL assay results indicated that Dp (100 μM, 12 h) was ineffective against certain human prostate cancer cells like Du145 and PC3 cells, but that it exhibited a dose-dependent apoptotic effect on LNCaP cells (p-53 positive). Treatment of LNCap cells with Dp led to enhanced expression of caspases-8, cleaved forms of caspases-3,−7, and PARP-1, whereas caspase inhibitors (zVAD and zDQMD) hindered the Dp-provoked reduction in cell viability. Inhibition of HDAC3 involved in transcriptional activities and upsurge in p53 expression by p53 oligomerization seem to play a role in Dp-induced apoptosis. Dp also showed synergistic effects with HDAC inhibitors, TSA, and MS-275 or HDAC3 siRNA by amplifying reduction in cell survival ability and enhancing the expression, acetylation, and oligomerization of p53 and expression of pro-apoptotic proteins p21 and Bax. Stimulation of the expression of wild-type HDAC3 and HDAC3D309A averted the effects of Dp on the expression of p53, p21, and Bax, whereas the C-terminal deletion mutant HDAC3ΔC did not interfere with the effects of Dp. Dp, during a 24-h treatment, stimulated the expression of p21, Bax, and Noxa in plasmid-transfected LNCaP cells, and the expression was reduced by HDAC3 and HDAC3D309A. Stimulation of caspases by Dp treatment resulted in cleavage of HDAC3, which provoked hyperacetylation and oligomerization of p53, and the expression p53 target genes (108). Furthermore, it was suggested that Dp arrests the signaling of β-catenin in PC3 cells (109).

#### Dp in Lung Cancer

Pal et al. evaluated the potential of Dp in therapy of non-small cell lung cancer (NSCLC). It was found that treatment of EGFR and VEGFR expressing NSCLC (NCI-H441 and SK-MES-1) with Dp (5–60 μM, 3 h) leads to downregulation of EGF- and VEGF-provoked and constitutive EGFR and VEGFR expressions, respectively. Dp, during a course of 48-h treatment, turned down PI3K/Akt signaling, and its downstream targets were activated by EGFR and phosphorylation of ERK1/2, JNK1/2, and p38 in the cell lines. The expression of Cyclin D1, PCNA, and anti-apoptotic proteins like Mcl-1, Bcl2, and Bcl-xL was suppressed, and the expression of pro-apoptotic genes like Bak and Bax was enhanced by Dp treatment. The expression of caspase-9 and−3 and cleavage of PARP were stimulated by Dp. An MTT analysis showed that Dp (5–100 μM, 48 h) reduced cell viability with IC_50_ values of 55, 58, and 44 μM for A549, SK-MES-1, and NCI-H441 cells, respectively. *In vivo* studies on male athymic nude mice (4 weeks old) implanted with NCI-H441 or SK-MES-1 cells demonstrated significant decline in tumor cell growth with Dp (1–2 mg) treatment. The expression of proliferation (Ki67 and PCNA) and apoptotic (caspase-3) markers was enhanced, and the expression of VEGF and CD31 was decreased in Dp-treated mice (110). Later on, it was found that Dp (10–40 μM) did not affect the growth and proliferation of lung cancer cell line (A549 or NCI-H460) but averted the CoCl_2_ (200 μM)- and EGF (20 ng/ml)-provoked expression of hypoxia-inducible factor-1α (HIF-1α) protein without altering the expression of HIF-1β in lung cancer, breast cancer (MCF-7), and prostate cancer (PC3M) cell lines. Rate of inhibition of HIF-1α was highest in A549 cells. RT-PCR revealed that Dp treatment also suppressed CoCl_2−_ and EGF-provoked VEGF (involved in angiogenesis) protein expression and lowered VEGF mRNA levels in all the above-mentioned cell lines. CoCl_2−_ and EGF-provoked hypoxia-response element (HRE) activity was hindered by Dp (10 μM). HIF-1α mRNA levels remained unaltered with Dp treatment, whereas MG132-stimulated HIF-1α was suppressed by Dp, indicating that Dp acts by interfering with the synthetic pathway of HIF-1α in A459 cells. CoCl_2_ and EGF treatment, within 10 min, led to elevation in the phosphorylation of ERK, PI3K, Akt, and mTOR, and P4-S6K, involved in regulation of HIF-1α expression, was brought down by Dp treatment (111).

Kang et al. showed that the combination of Dp (5 μM) and ionizing radiation (4 Gy/min) have better outcome than either agent alone. Dp exerted its beneficial effects *via* inducing autophagy and activating JNK/MAPK pathways to enhance apoptotic cell death in human lung cancer cells (A549) (112).

#### Dp in Skin Cancer

It has been known that cyclooxygenase-2 (COX-2) is an important target for developing anticancer therapy, as it is overexpressed in (abnormal and elevated levels) skin cancer. Pretreatment with Dp (5–20 μM, 1 h) has been found to hinder UVB-provoked COX-2 and prostaglandin E_2_(PGE_2_) expression in the JB6P+ epidermal cell line of female ICR mice. However, at this concentration, Dp was not able to affect the survival ability of cells. WBA data revealed that Dp down-regulated the expression of UVB-provoked MAPKK4, JNK, c-Jun, p38, ERK1/2, p90RSK, AP-1, NF-κB, and several downstream substrates of PI3K pathway, but the expression of MAPKK-3/6, and 7, and MEK-1/2 remained unaffected. *In vitro* and *ex vivo* kinase analyses proved that Dp inhibited the expression of MAPKK4 and PI3K. Pull-down assays and molecular modeling data revealed that Dp is bound to the ATP binding site of MAPKK4 and PI3K. The ability of Dp to hinder MAPKK4 and PI3K expression is related to downregulation of COX-2 expression in JB6P+ cells (113).

Delphinidin (Dp) (20 or 40 μM) pre-treatment of JB6 P+ cells also inhibits TNF-α and provokes the expression of COX-2 promoter and COX-2. Dp inhibits TNF-α more effectively than other phenolics like resveratrol (40 μM) or gallic acid. Dp (40 μM) treatment led to reduction in the expression of TNF-α-induced downstream kinases involved in COX-2 expression like AP-1 by 82% and NF-kB by 44%, and several upstream kinases like JNK, p38 MAP, Akt, p90RSK, MSK1, and ERK. A pull-down assay demonstrated that Fyn kinase expression was also suppressed by Dp treatment. It was proved that Dp binds with Fyn kinase irrespective of ATP concentration. Molecular docking highlighted the interaction between Dp and Fyn kinase, as Dp forms hydrogen bonds with the side chain of Gln161 and the backbone carbonyl group of Met249 in the SH2 domain, with the backbone carbonyl group of Tyr343 in the catalytic kinase domain, and showed hydrophobic interaction with Ile402 and Met344 of the kinase domain. Dp also suppressed FAK (a Fyn kinase downstream protein) (114).

Kuo et al. demonstrated that treatment of JB6 P+ cells with Dp (5, 10, and 20 μM) for 1, 3, or 5 days can significantly reduce cellular proliferation and enhance cell viability above 50%. Dp pretreatment of JB6 P+ cells led to reduction in TPA-induced anchorage-independent growth by 69.4, 74.4, and 99.4% with increase in concentration of Dp from 5 to 20 μM. Dp enhanced normalized relative luminescence and antioxidant responsive element (ARE)-dependent luciferase activity in a dose-dependent manner. Furthermore, Dp escalated the expression of Nrf2 and its target genes, which, when bound to the ARE region, triggered the expression of the carcinogen-detoxifying phase 2 enzymes HO-1 and NQO1 and the reactive oxygen species (ROS) scavenger SOD1, and led to CpG demethylation in in the Nrf2 promoter. It was suggested that Dp exhibits its activity by lowering the expression of DNMTs (DNMT1 and DNMT3a) and class I and class II HDACs, which further led to decreased CpG methylation (115).

#### Dp in Osteocarcinoma

The role of Dp has also been investigated in osteocarcinoma. Interestingly, Dp, in a dose-dependent manner, decreased cell viability by arresting the proliferation of osteosarcoma cell (OS) line (U2OS) and by provoking reactive oxygen species (ROS) production. A western blot analysis indicated that Dp also provoked the expression of LC3-II gene, caused degradation of p62 protein, and led to autophagosome formation. Enhanced activity against OS cells was observed on pre-treatment with the combination of Dp and autophagy inhibitors (3-MA and bafilomycin A1). Flow cytometry and a cell cycle analysis showed that Dp treatment enhanced DNA content in the sub-G_1_ phase and cell number in the G_2_/M phase along with reduction in cell number in the G_0_/G_1_ phase (116).

Delphinidin (Dp) treatment of OS cell lines (U2OS, HOS, and MG-63) reduced the survival ability of U2OS and HOS cells. A colony-forming assay conducted on U2OS and HOS cells revealed that Dp (0.1–10 μM, 7 days) significantly hindered growth and proliferation. Treatment of U2OS and HOS cells with Dp (75 μM, 6–24 h) showed elevation in condensation of nuclear ratio by 48 and 37%, respectively; shrinkage of tumor cells and apoptosis were also observed in a time-dependent manner. The expression of proteins involved in apoptosis, like Bcl-2, was reduced, and the expression of Bak, pro-caspase-2, and PARP was stimulated, therefore enhancing the release of Cyp c by Dp treatment in OS cell lines. A transwell chamber assay revealed that Dp (75 μM, 24 h) averted metastasis in OS cells. WBA demonstrated that the expression of EMT markers involved in tumor metastasis like E-cadherin was enhanced, while that of N-cadherin, Snail, and Slug was reduced by Dp. The expression of p38 and ERK 1/2 was also hindered by Dp. Dp behaved synergistically with ERK1/2 and p38 inhibitors (SB203580 and PD38059, 20 μM), as co-treatment enhanced the expression of E-cadherin, while that of N-cadherin, Snail and Slug was reduced to a greater extent compared to Dp alone, signifying that Dp prevents metastasis in OS cells by downregulating the ERK/MAPK signaling pathway (117).

#### Dp in Miscellaneous Cancers

Increased expression of human glyoxalase I (an enzyme involved in detoxification of methylglyoxal, which is highly reactive and involved in apoptosis) has been demonstrated in many tumors such as those of the colon, prostate, and lungs. According to an *in vitro* GLO I assay, among anthocyanidins, Dp was the most dominant inhibitor of recombinant GLO I, with an IC_50_ value of 1.9 μM, and 11.7 and 16.4 μM for cyanidin and pelargonidin, respectively. Strong hydrogen bonding was observed between the three hydroxy groups in the B ring of Dp and amino acids (Asn103B, Arg122A, and Arg37B) of GLO I in humans. Dp hindered the growth and proliferation of HL-60 cells in a time-dependent manner at an interval of 24 and 48 h and IC_50_ values of 80 and 40 μM, respectively. GLO I expression suppressed by Dp led to increase in concentrations of toxic methyl glyoxal, which led to apoptosis (118).

In an interesting study, treatment of human leukemia cell line (HL-60) with Dp at minimal doses resulted in cell death within a 24-h period. However, treatment of hepatocellular carcinoma (HCC) cell lines (SMMC7721, HCCLM3, and MHCC97L) with Dp and Cy3R proved to be insignificant, as Dp was not able to stimulate apoptosis. However, treatment with Dp and Cy3R led to formation of vacuoles within HCC cells, which was related to their free radical scavenging activity and autophagic degradation. Dp was unable to provoke endoplasmic reticulum (ER) stress, as the expression of proteins like ATF-4, CHOP, and Bip remained unaffected. 3-methyladenine- (autophagy inhibitor) and bafilomycin A1-averted Dp stimulated cellular vacuolization, confirming the cause to be autophagic degradation. Results from an immunoblot assay confirmed the enhanced expression of LC3-II protein and supported the fact that Dp can induce autophagy in SMMC7721 cells. Co-treatment with Dp and 3-methyl adenine effectively induced necrosis (50% cell death observed in a 48-h period) in HCC cells, and no caspase activity was recorded, suggesting no apoptotic cell death (119).

Lim et al. investigated the role of Dp in inhibiting EGF-induced epithelial-to-mesenchymal transition (EMT) in HCC. Dp was noted to exhibit dose-dependent anti-proliferative effects against Huh7 and PLC/PRF/5 cells. It also inhibited EGF-induced morphological changes from epithelial to mesenchymal s in HCC primarily by inhibition of EGFR/AKT/ERK signaling pathway (120).

Kang et al. demonstrated the usefulness of Dp in treatment of urinary bladder cancer. Dp showed promising anti-proliferative actions against T24 cell lines. Dp produced a dose-dependent cytotoxic effect (IC_50_ of 34 μg/ml) by apoptosis induction and ROS generation in cancer cells (121).

### Synergistic Anticancer Effects of Dp

Dp acts synergistically with a variety of well-known anticancer agents. Synergistic anticancer effects have been observed with Dp and some drugs. The combination of Dp, cisplatin, and paclitaxel enhanced cytotoxicity by 50%, and Dp also boosted the activity of cisplatin against ES2 cells. The combination of Dp (10 μM) and paclitaxel (20 μM) proved to be more effective against SKOV3 cells than paclitaxel alone (101, 102). Treatment of human glioblastoma cell lines (U87MG and LN18) with Dp (50 μM) alone had minor effect on cell survival ability, whereas synergism was observed in the combination with miR-137. A Matrigel layer assay showed that individually AzaC, Dp, and miR-137 mimics suppressed metastasis and cell invasive ability in U87MG and LN18 cells, and that treatment with miR-137 and, subsequently, with Dp proved to be most significant. A flow cytometric analysis showed that glioblastoma cell lines treated with combination of miR-137 and Dp exhibited characteristic features of apoptotic cells and enhanced expression of annexin V (122, 123). The combination of Dp (8 μM) and As (III) (0–20 μM) resulted in enhanced cytotoxic effects against HL-60 cell lines with reduction in IC_50_ value from 11.2 to 1.5 μM in HL-60 cells, 2.4 to 1.4 μM in NB4 cells, and 9.9 to 8.2 μM in PMBCs (124). Synergism was also noted in the combination of Dp (0–30 μM, 12 h) and TRAIL (50 ng/ml), as the combination stimulated PARP cleavage in TRAIL-sensitive (Du145) and resistant (LNCaP) cells. The cleavage of caspase-3/7 and expression of caspase-8.9 were enhanced by the combination treatment and, in the presence of zVAD (40 μM), a caspase inhibitor, the apoptotic activity of the combination was significantly reduced. The expression of DR5, BAX, p21, and p53 was enhanced, and the mRNA expression of XIAP, cIAP-2, Bcl-2, survivin, and MCL-1 was decreased by treatment with the combination, whereas inhibition of the expression of DR5 and Bax by siRNA treatment led to aversion of Dp-stimulated TRAIL-provoked caspase-3 activation in LNCap and Du145 cells. HDAC3 (which modulates transcriptional activities and whose inhibition promotes PARP cleavage) expression (confirmed by siRNA inhibition of HDCA3) was suppressed by the combination rather than Dp or TRAIL alone (125).

### Anti-inflammatory Activity

Anthocyanins, in particular Dp, possess a broad range of biological activity, especially anti-inflammatory effect. Dp is reported to be a specific histone acetyltransferase (HAT) inhibitor of p300/CBP acetyltransferase and effective in ameliorating symptoms associated with rheumatoid arthritis (RA). It has also been documented that histone deacetylase, histone methyltransferase, and sirtuin 1 activities are unaffected by Dp treatment. Dp, at a high concentration (100 μM), also averted cell viability (30%) in human RA synovial cell line (MH7A), and downregulated the mRNA and protein expressions of NF-κB p65 subunit and decreased cytokine production by inhibiting TNF-α and provoking p65 expression (126).

Dp-3S, a major anthocyanin present in dried calyces of *Hibiscus sabdariffa* L and Dp (100 μM) were shown to hinder LPS-provoked NO and iNOS production, and expression of inflammatory cytokines like TNF-α, IL-6, and MCP-1 in a dose-dependent manner. Dp (aglycone) exhibited a more prominent activity than Dp-3S and was able to reverse the inflammation caused by NF-κB and MEK/ERK signaling in RAW264.7 cells (20).

Dp is also effective in treatment of spinal cord injury (SCI)-induced inflammation in an SD rat model with depleted Basso, Beattie, Bresnahan (BBB) score. Dp at a dose of 200 mg/kg treatment for 21 days resulted in elevation of BBB scores and subsequent lowering of intramedullary spinal pressure compared to a control group. The anti-inflammatory effects could be due to significant drop in COX-2 and PGE_2_ level (127).

### Protection Against Nasal Polyps

Nasal polyps are non-cancerous benign lesions arising from the mucosa of nasal sinuses or nasal cavity characterized by extracellular matrix (ECM) accumulation. Nasal polyp-derived fibroblast, upon treatment with Dp (0–20 μM), showed suppression of the mRNA expression marker of myofibroblasts (α-SMA) and extracellular matrix proteins (collagen and fibronectin) (128, 129). In human airway epithelial cells, Dp inhibits the expression of MUC8 and MUC5B by acting through toll-like receptor (TLR4)-mediated ERK1/2 and p38 MAPK signaling pathways. Thus, the data support the effectiveness of Dp in the inflammatory airway diseases; therefore, Dp can be considered a promising lead for future research (130).

### Neuroprotective Activity

Neurological diseases, specifically Alzheimer's and Parkinson's, are directly correlated to oxidative stress. Dp exhibits neuroprotective activity against hypoxia (14). It has the ability to attenuate the oxidative stress of H_2_O_2_ in SK-N-SH cells by inactivation of the ASK-JNK/p38 signaling pathway (14, 131). Dp also showed an effective response by abrogating intracellular calcium influx and tau phosphorylation against cytotoxicity induced by Aβ_25−35_ (14).

The 3-O-β-galactoside of Dp has the ability to cross the blood-brain barrier (BBB) and its presence was detected in various regions of the brain in experimental animals, indicating its potential in treatment of various disorders related to the brain. Dp, at different concentrations (4, 20, and 100 μg/ml) could attenuate neurotoxicity stimulated by the administration of amyloid beta (Aβ) in PC12 cells. Pretreatment with Dp (20 μg/ml) was also shown to reduce the Aβ-induced stimulation of phosphorylated GSK-3β and tau (elevated levels involved in pathogenesis of Alzheimer's disease) levels (79, 132). Radio ligand binding assays demonstrated that Dp has the potential to bind to human CB1 and CB2 with K1 and K2 value of 21.3 and 34.3 μM, respectively (14).

Treatment with Dp for 25 days in rats with lesions of nucleus basalis of Meynert (NBM) led to normalization of body weight. In a Morris water maze test, Dp-treated rats reached the hidden platform 3 times faster than rats with NBM lesions, signifying better spatial memory, movement control, and cognitive mapping. Furthermore, significant reduction in ROS and AChE activity and levels of amyloid precursor protein (APP) and Aβ protein of rats with NBM lesions was noted after Dp treatment in the hippocampal area. Molecular docking studies confirmed that Dp bound to the active site of AChE and to Aβ protein with a binding energy of −8.11 and −9.43 Kcal/mol, respectively. A decline in deposits of Aβ plaque in Dp-treated rats was also noted because of efficient binding of Dp with the Aβ protein (133).

### Cardioprotective and Antihypertensive Activity

Angiotensin-converting enzyme (ACE) is an important component of renin-angiotensin system (RAS), which is involved in regulation of blood pressure. Anthocyanin-rich fractions extracted from *Hibiscus sabdariffa* hindered ACE activity, with an IC_50_ value of 91.2 μg/ml. The two major anthocyanins, *viz*., Dp-3-S and Cy-3-S, were also found to be effective in suppressing ACE activity, with IC_50_ values of 84.55 ± 2.2 and 68.41 ± 2.87 μg/ml, respectively. A kinetic analysis revealed that the anthocyanin Dp (31.9 μM) was the more effective compound than Cy-3S (56.9 μM), which in turn was more significant than anthocyanin-rich fractions (K*i* = 0.065 mg/ml). Dp, because of presence of 3 -OH and Cy with 2 -OH groups in B ring, exhibits prominent interaction with active sites of ACE (134).

Dp, Cy, and quercetin, at a dose of 100 μM, exhibited an ACE inhibitory activity comparable to that of a clinically used ACE inhibitor, captopril (10 μM), in human embryonic kidney (HEK)-293 cells. Pretreatment with Dp, Cy, and quercetin was also able to avert the steroid-induced elevation of ACE levels. RT-qPCR data revealed that the mRNA expression of ACE and renin was suppressed by Dp, Cy, and quercetin, and that captopril enhanced renin mRNA expression. Dp and Cy also inhibited the ACE protein expression in HEK-293 cells. Angiotensin II-provoked AT1R internalization was unaffected by pretreatment with Dp, Cy, and quercetin. On the other hand, losartan inhibited it, signifying that these compounds do not interfere with the angiotensin II receptor (AR) pathway (135).

Administration of Dp (15 mg/kg/day) for a period of 8 weeks in mice was shown to avert cardiac hypertrophy, oxidative stress, and cardiac dysfunction with no signs of toxicity. Furthermore, Dp was noted to reduce myocardial fibrosis and controlled the enhanced mRNA levels of collagen I, collagen III, and connective tissue growth factor (CTGF) in the cardiac extracellular matrix induced by high Ang II levels caused by TAC. Activation of AMPK by Dp led to hindrance in the expression of NOX subunits (p47phox) and, therefore, reduction in Ang II-induced cardiomyocyte hypertrophy. Upregulated levels of Erk1/2, Jnk1/2, and p38 due to TAC were controlled by Dp administration, signifying the role of Dp in MAPK pathway. In aged mice, Dp demonstrated to be effective in reducing the visible characteristics of aging and incidence of cardiac hypertrophy by reduction in superoxide production and NOX activity, and regulation of the AMPK/NOX/MAPK signaling pathway (136).

### Antidiabetic Activity

Anthocyanins potentially modulate carbohydrate metabolism and blood glycemic levels, and help reduce many cardiovascular risk factors (137). Dp-3-rutinoside (Dp-3-R) has the ability to increase glucagon-like peptide-1 (GLP-1) secretion in GLUTag cells mediated through the Ca^2+^/calmodulin-dependent Ca^2+^-CaMKII pathway. It has been reported that the presence of 3 hydroxyl groups or two methoxyl moieties in the aromatic ring of Dp-3-R is important to stimulate GLP-1 secretion (138). Streptozotocin (STZ)-induced diabetes in mice (male BALB/c), upon treatment with Dp (100 mg/ml), in free and liposomal forms for 8 weeks displayed reduction in albumin glycosylation rate, and the data revealed that the liposomal form of Dp could be developed as an effective treatment modality to control diabetes (87).

Oral pre-administration of black currant extract (5 mg/kg) containing 1 mg Dp-3-R/kg in STZ-induced diabetes in rats showed decrease in blood glucose levels at 30- and 60-min intervals, rise in serum insulin, and elevation in GLP-1 level at 15- and 30-min intervals after IP glucose (2 g/kg) injection. An HPLC analysis revealed that nonsignificant concentrations of Dp-3R degradation products like Dp, gallic acid, and phlorogucinol aldehyde were present in the GI tract. Furthermore, an analysis demonstrated that individual administration of Dp-3R degradation products (Dp, GA, and PGA) was not able to stimulate GLP-1 secretion in GLUTag cells, indicating that the anthocyanin form of Dp-3R present in black currant extract is responsible for controlling blood glucose levels, and that it is not degraded until after 45-60 min of black currant extract administration (139). Furthermore, administration of Dp-3R rich black currant extract along with high sucrose diet for a period of 2–7 weeks to type-2 diabetic mice (male kk-A^y^) resulted in significant decrease in serum glucose concentration and increase in basal GLP-1 levels because of enhanced mRNA and protein expression of PC1/3 in the ileum (140).

Human interventional studies also concluded that high doses of anthocyanins have a potential in prevention and management of type 2 diabetes and further warrant deeper analyses to claim their benefits for use in human (137). Dp protected pancreatic beta cells against high glucose-induced injury by increasing the phosphorylation of AMPK alpha: Thre172 stimulated glucose uptake by cells (141). Delphinol^®^, a product that contains 25% Dp and 35% total anthocyanins, has the ability to decrease post-prandial glucose and insulin in clinical trial. It significantly decreased basal glycemia and insulinemia in a dose-dependent manner, and resulted in improvement in blood glucose level when administered to patients with pre-diabetes (137).

### Anti-osteoporotic Activity

Osteoclast precursors (RAW 264.7 cells), upon treatment with anthocyanins (Cy, Dp, and Pg at a dose of 0.25–20 μg/ml) extracted from bilberry and blackcurrant, exhibited significant decrease in RANKL (which stimulates osteoclast formation) expression and osteoclast formation.

*In vivo* X-ray data highlighted that Dp prevented bone deterioration in ovariectomized female C57BL/6 mice with sRANKL-induced osteoporosis. Dp, at a small dose of 3 mg/kg, inhibited bone resorption, and uterus weight remained unchanged, signifying that Dp acted in a different manner. A TransAM assay proved that Dp and other anthocyanins act by arresting the activation of NF-κB pathway and reducing the expression of c-fos, Nfac, MMP9, and Trap genes involved in the formation of osteoclasts. Dp is reported to be the most potent inhibitor of osteoclast differentiation and considered as an effective agent for preventing bone loss in women with postmenopausal osteoporosis (142).

Dp-3R, isolated from *Solanum melongena* L., was also reported to enhance the cell viability of an osteoblast precursor cell line (MC3T3-E1). Dp-3-R (10^−9^ M) increased the expression of Co11A1, ALP, and OC (involved in osteoblast differentiation). Dp-3-R also simulated the expression of β-catenin, showing a positive feedback in the Wnt/β-catenin signaling pathway (143). Another report concluded that Dp possesses an antiproliferative and apoptosis effect in human osteosarcoma HOs and U2OS cells. It has also been highlighted that Dp suppresses cell migration and prevents abrogation of the MAPK signaling pathway (117).

Dp has the ability to activate cytoprotective autophagy in order to protect chondrocytes against H_2_O_2_-induced oxidative stress *via* activation of Nrf2 and NF-KB. Hence, it plays a major role in critical management of osteoarthritis (OA) and helps in preventing the development and progression of OA (144). Pretreatment with Dp-3-O-β-D-glucoside chloride (20 μg/ml) and Dp (20 and 40 μg/ml) has been shown to reduce osteoclast formation in RANKL-treated embryos of medaka (145).

### Role in Skeletal Muscle Atrophic Activity

An effect of Dp on muscle atrophy has been reported. Dp has the ability to effectively supress mechanical unloading-induced muscle weight loss. Dp treatment was able to reverse muscle atrophy induced by tail suspension method in C57BL/6 J mice. Dp also prevented the enhanced expression of genes involved in antioxidation, redox regulation, and ROS, ubiquitin ligase, and protein degradation in atrophic mice. Elevated expression of Cbl-b (RING-type ubiquitin ligase, elevated in muscle atrophy) in C2C12 myotubes due to dexamethasone (glucocorticoid) was also controlled by Dp treatment (146). A further study showed that Dp treatment leads to decline in MuRF1 (involved in muscle protein degradation *via* ubiquitination) mRNA, and protein expression was induced because of dexamethasone treatment in C2C12 cells. It was hypothesized that Dp prevents muscle atrophy by enhancing miR-23a and NFATc3 expression (147).

### Anti-psoriatic Activity

Dp at a dose of 80 μM, significantly suppresses the proliferation of normal human epidermal keratinocytes (NHEK) and then initiates apoptosis. However, at a dose of 10–40 μM, Dp did not alter the expression of genes involved in the apoptotic pathway. A 3D epidermal equivalent model showed that Dp treatment enhanced the expression of caspase 14 and keratin 1 (148).

Topical application of Dp (0.5 and 1 mg/cm^2^ skin area) on psoriasis from lesions present on the flaky skin of mice significantly decreased the lesions and provoked the expression of proteins that are downregulated in psoriasis like caspase-14. Dp-treated mice exhibited reduction in infiltrating macrophages around the epidermis and prevented protein production and mRNA expression of various inflammatory cytokines like TNF-α (149). A 3D human psoriatic skin-equivalent PSE (SOR-300-FT) was treated with Dp for a period of 48–72 h, which resulted in induction of cornification and reduced epidermal thickness with extended treatment (5 days) comparable to vitamin D3 treatment. Levels of pro-inflammatory cytokines like IL-1α, IL-1β, IL-6, IL-8, IL-10, and TNF-α were also decreased by Dp. However, vitamin D3 and retinoic acid only affected IL-1α and IL-1β (150). Histopathological studies demonstrated that Dp decreases acanthosis, epidermal rete ridge projections into the dermis, micro abscesses, and epidermal thickness in imiquimod (IMQ)-treated mice. Dp also impeded the expression of Akt and p70S6K, suggesting the effectiveness of Dp treatment in an IMQ-stimulated psoriasis model (19).

*In silico* docking studies revealed binding efficiency with PI3Kinases. Dp also exhibited good affinity for p70S6K. Dp was found to interact with rapamycin binding site, FRB of mTOR, with BE-9 kcal/mol better than rapamycin. Dp also attenuates the expression of IL-22 and stimulates the expression of PI3Ks (p110 and p85), Akts (Ser473 and Thr308), mTORs (Ser2448 and Ser2481), PRAS40, and p70S6K (Thr389) (19).

### Anti-hepatotoxic Activity

Dp promotes Nrf2 nuclear translocation and leads to increase expression of antioxidant protein HO-1, which is an Nrf2-related phase II enzyme heme oxygenase-I present in HepG2 cells. This confirms the hepatoprotective effect of Dp and its role in regulation of the expression of Nrf2/HO-1 to protect HepG2 cells (151). Dp has been demonstrated to decrease hepatic inflammatory markers such as TNF-α, IL6, and INF. It also decreased the immunopositivity of nuclear factor kappa-B (NF-κB) and CYP2E1 in liver tissues and restored altered hepatic architecture (152). Dp attenuated CCl_4_-induced hepatotoxicity in Balb/C mice and significantly controlled elevated serum levels of alanine aminotransferase (ALT), aspartate aminotransferase (AST), and alkaline phosphatase (ALP), and improved cleaving cholinesterase (ChE) activity. Increase in liver weight, hydroxyproline content, and oxidative stress (reduced GSH/GSSG ratio) due to CCl_4_-induced hepatotoxicity was also normalized by Dp. Histopathology studies confirmed that Dp (10 mg/kg)-treated mice have reduced collagen deposits, hepatic lesions, and hepatocyte ballooning, and decreased necrosis compared to a control group. Mice treated with 25 mg/kg treatment showed significant reduction in hepatic fibrosis. The expression of α -SMA (marker for hepatic fibrosis) in activated hepatic stellate cells and the hepatic expression of TNF-α and TGF-β1 were inhibited by Dp treatment, while MMP-9 and metallothionein I/II expression was enhanced by Dp treatment (153).

### Anti-viral Activity

Dp is considered as a new inhibitor of hepatitis C virus (HCV) entry in a pangenotypic manner by acting directly on viral particles and impairing their attachment to the surface of cells (154). It also inhibits the expression of viral RNA in a dose-dependent manner (1–10 μM). It has also been observed that Dp acts in early stages of viral infection. Furthermore, an investigation revealed that Dp does not act by interfering with endosomal pH. Dp was also found to be highly virucidal against the DENV-2 and African strains (MR766) of ZIKA virus compared to the American strain (PA259459) (155). Its role in viral replication is considered as a prominent pharmacological action; hence, researchers are using it as a potential lead for the severe acute respiratory syndrome-coronavirus 2 (SARS-CoV2) main protease by computational approaches, especially structure-based virtual (SBV) screening (156). Wu et al. (157) screened 480 bioactive phenolic compounds; among those identified, Cy-3R and Dp-3R were considered as potential inhibitors of RNA-dependent RNA polymerase (RdRp) in SARS-CoV2. In fact, the binding energy of Cy-3R (−107.8 kcal/mol) and Dp-3R (−90.70 kcal/mol) was much better than that of remdesivir (−55.0 kcal/mol) (157).

## Granted Patents

Patients has been granted for PD pertaining to innovation in isolation technique and cosmetic and therapeutic uses. Dp is patented to combat melanoma cells, as an immunosuppressant ingredient in formulation, as an anti-infective agent, and for preventing hair loss, etc. Details of various patents granted for Dp are presented in Supplementary Table 1.

## Summary, Challenges, and Future Delphinidin Research

Dp is one of the major and bioactive plant pigments with six hydroxy groups present in the flavylium ion. It occurs in both anthocyanin and anthocyanidin forms in berries (blueberry, billberry), concord grapes, blackcurrant, roselle, some tropical fruits, vegetables (eggplant), roots, cereals and wine. Dp (anthocyanidin) is found in nature linked to a variety of sugar moieties, such as glucose, arabinose, galactose, and sambubiose, in the C-3 position (anthocyanin form). Although Dp is more active in its anthocyanidin form, the presence of sugar moiety in the 3rd position in ring C is also vital for its bioavailability. Because of its wide therapeutic spectrum, it has been used along with other nutraceuticals and as a dietary supplement. A brief summary of the reviewed literature in this article highlights the biosynthesis, laboratory synthesis, and stability profiling of Dp. Various glycosides of Dp are extracted in combination with a variety of anthocyanins by a number of extraction and purification techniques; isolation, analysis, and characterization of Dp have been performed using semi-preparative HPLC, UPLC, UV-visible spectroscopy, ESI-MS, IR, and NMR analytical techniques. Several patents have been granted for Dp research that are related to its (i) synthesis, (ii) isolation and analysis, (iii) use in cosmetics, and (iv) therapeutic use as anticancer, antimicrobial, and immunosuppressant, etc. Findings on biosynthetic pathways, chemical synthesis, isolation, and quantitative analysis techniques presented in this review will open pathways and guide researchers to conduct more significant studies on Dp to improve its utility in the cosmetic, food, and pharmaceutical industries.

Dp is highly stable under acidic conditions but unstable under intestinal and neutral pH conditions. Dp, upon thermal degradation and under alkaline stressed conditions, produces gallic acid and phloroglucinaldehyde. Dp suffers from low oral bioavailability compared to other bioactive polyphenolic secondary metabolites such as anthocyanidins and flavonoids. Low bioavailability reduces its clinical utility and, therefore, poses a major challenge to the scientific community. However, efforts have been made to improve its bioavailability as well as stability under alkaline conditions through complexation with cyclodextrins and/or by preparing microencapsulation and nano-formulations for optimized delivery *in vivo*. Its highly stable analogs prepared by simple substitutions and advanced liposomal delivery protocols are reported to be successful. In spite of these efforts, an ideal Dp formulation with high bioavailability and high stability is not in sight. Another problem with Dp usage is its ability to inhibit CyP450, which increases chances of drug interactions especially in patients taking medications for chronic diseases.

Dp is being recognized as a potent lead in the discovery and development of therapies for treatment of a variety of critical health conditions such as COVID-19. Intensive investigation is ongoing to unlock the hidden potential and diverse biological activities of Dp beyond its usage as a natural pigment, especially the broad spectrum anticancer and antiviral activities. Findings of various studies have demonstrated that Dp in both forms, *viz.*, anthocyanidin and anthocyanin, exhibits promising therapeutic anticancer activity against a variety of cancers such as breast, ovarian, colon, prostate, lung, hepatic, bone, blood, and skin cancers. Furthermore, Dp also exhibits synergistic effects when used in combination with some clinically used anticancer drugs. Recently published studies have also unveiled Dp as a potent cardioprotective agent and very effective against psoriasis, osteoporosis, and a variety of viral species. Some of the mechanisms involve are scavenging of free radicals; interfering with protein targets of the PI3K/Akt/mTOR, MAPK, and ubiquitin-proteasome pathways; lowering the expression of NOX, cytokines, mucins, MMPs, and STATs; inhibiting of ACE; hindering the entry of viral particles; and enhancing the secretion of insulin, GLP-1, LCE3 genes, and certain epidermal proteins. Although the anticancer potential and other useful bioactivities of Dp have been evaluated and well proven in preclinical studies (*in vitro* experimental studies and *in vivo* animal studies), there is scarcity of human interventional studies. Preclinical data from these studies are quite encouraging; therefore, this compound deserves further evaluation in clinical trials.

Several studies have also shown Dp to exert beneficial effects on the gut microbiome population. It promoted the proliferation of oxygen-sensitive bacterial population by decreasing oxygen tension in gut lumens. It also significantly enhanced the proliferation of *Bifidobacterium spp*. and *lactobacillus-Enterococcus spp*., and inhibited the growth of a species of pathogenic bacteria, *Clostridium histolyticum*. However, these effects were observed in only few *in vivo* and small human interventional studies; therefore, it cannot be generalized that supplementation with Dp leads to favorable effects. Hence, there is a need to conduct well designed studies to evaluate the beneficial effects of consumption of anthocyanin-rich food including Dp on the proliferation of healthy anaerobic gut microbiota along with mechanistic studies.

The meticulous investigation of various published literature confirmed the fact that Dp in its anthocyanidin and anthocyanin forms is a promising therapeutic candidate for scientific research beyond nutrition. However, because of lack of proper clinical data, the true potential of Dp is yet to be established.

## Author Contributions

AH, SK, UMD, MA, AAA, and AA significantly contributed in the preparation of the manuscript. SK and UMD prepared the figures and tables. AH and SK equally contributed to complete the chemistry part of the manuscript. HC and AA did the revision of the manuscript. All authors read and approved the final manuscript.

## Funding

This research study was funded by Institutional Fund Projects under Grant No. IFPRP:578-156-1442. Therefore, the authors gratefully acknowledge the technical and financial support from the Ministry of Education and King Abdulaziz University, Jeddah, Saudi Arabia.

## Conflict of Interest

The authors declare that the research was conducted in the absence of any commercial or financial relationships that could be construed as a potential conflict of interest.

## Publisher's Note

All claims expressed in this article are solely those of the authors and do not necessarily represent those of their affiliated organizations, or those of the publisher, the editors and the reviewers. Any product that may be evaluated in this article, or claim that may be made by its manufacturer, is not guaranteed or endorsed by the publisher.

## Abbreviations

ALP: alkaline phosphatase
ALT: alanine aminotransferase
AST: aspartate aminotransferase
AMPK: AMP-activated protein kinase
Bcl2: B-cell lymphoma 2
BE: binding energy
BrdU: bromodeoxyuridine/5-bromo-2'-deoxyuridine
Caspases: cysteine aspartyl-specific proteases
CCK: 8-cell counting kit-8
ChE: cholinesterase
COX-2: cyclooxygenase-2
DAD: diode array detector
DCF: 2 Fde array detectorecific prote
Dp: delphinidin
ELISA: enzyme-linked immunosorbent assay
EMSA: electrophoretic mobility shift assay
ERKs: extracellular-signal regulated kinases
ESI-MS: electrospray ionization mass spectrometry
FACS: fluorescence-activated cell sorting
GSH: glutathione
GSSG: oxidized glutathione
HDAC: histone deacetylase
HER2: human epidermal growth factor receptor-2
HGF: hepatocyte growth factor
HPLC: high-performance liquid chromatography
IFN-γ: interferon gamma
IL: interleukin
iNOS: inducible nitric oxide synthase
IR: infrared spectroscopy
LPS: lipopolysaccharide
MAPK: mitogen activated protein kinase
MMP: matrix metalloproteinase
MMT- 3-(4, 5-dimethylthiazol-2-yl)-2: 5-diphenyl tetrazolium bromide
MTS, [3-(4: 5-dimethylthiazol-2-yl)-5-(3-carboxymethoxyphenyl)-2-(4-sulfophenyl)-2H-tetrazolium]
mTOR: mammalian target of rapamycin
NF-ma: nuclear factor-t
NMR: nuclear magnetic resonance
NOX: NADPH oxidase
OPG: osteoprotegerin
PARP: poly(ADP-ribose) polymerase
PCNA: proliferating cell nuclear antigen
PGE2: prostaglandin E2
PI3K: phosphoinositide-3 kinase
qPCR: quantitative polymerase chain reaction
RANKL: receptor activator of nuclear factor-nophenyl)
ROS: reactive oxygen species
RT-PCR: reverse transcription polymerase chain reaction
SBV: structure-based virtual screening
SRB: sulforhodamine B
STZ: streptozotocin
t-BHP: tert-butyl hydroperoxide
TEM: transmission electron microscopy
TNF-α: tumor necrosis factor alpha
TQ: triple quaropole
TUNNEL: terminal deoxynucleotidyl transferase dUTP nick end labeling
UPLC: ultra performance liquid chromatography
UV: ultraviolet
VEGF: vascular endothelial growth factor
WBA: Western blot analysis.
