# Supplementary material for: Chemistry and Pharmacological Actions of Delphinidin, a Dietary Purple Pigment in Anthocyanidin and Anthocyanin Forms
Source: Front Nutr. 2022 Mar 17;9:746881. doi: 10.3389/fnut.2022.746881 (PMC8969030; doi:10.3389/fnut.2022.746881)
Supplement: Supplementary file 1 [file Data_Sheet_1.pdf]

Supplementary material

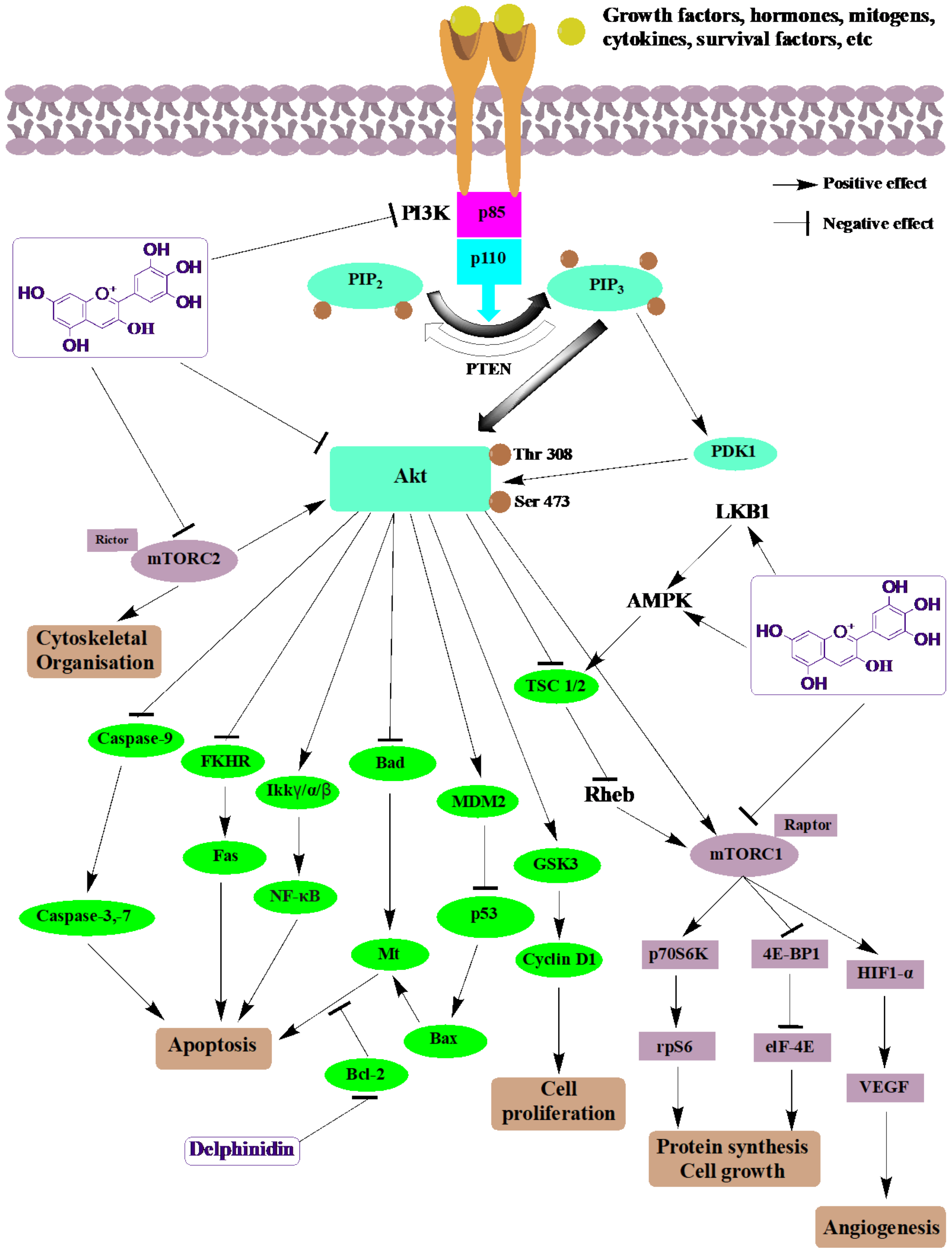

Figure S1. Schematic representation of the effect of Delphinidin on the PI3K/Akt/mTOR signaling pathway. Dp promotes apoptosis, autophagy and prevents cell growth, proliferation, angiogenesis, metastasis in the cancer cells by stimulating the expression of various targets genes like Caspases, Bax

AMPK, LKB1 and inhibiting the expression of PI3K, Akt, NF-κB, Bcl-2, mTORC1, mTORC2 involved in the regulation of the PI3K/Akt/mTOR signaling pathway.

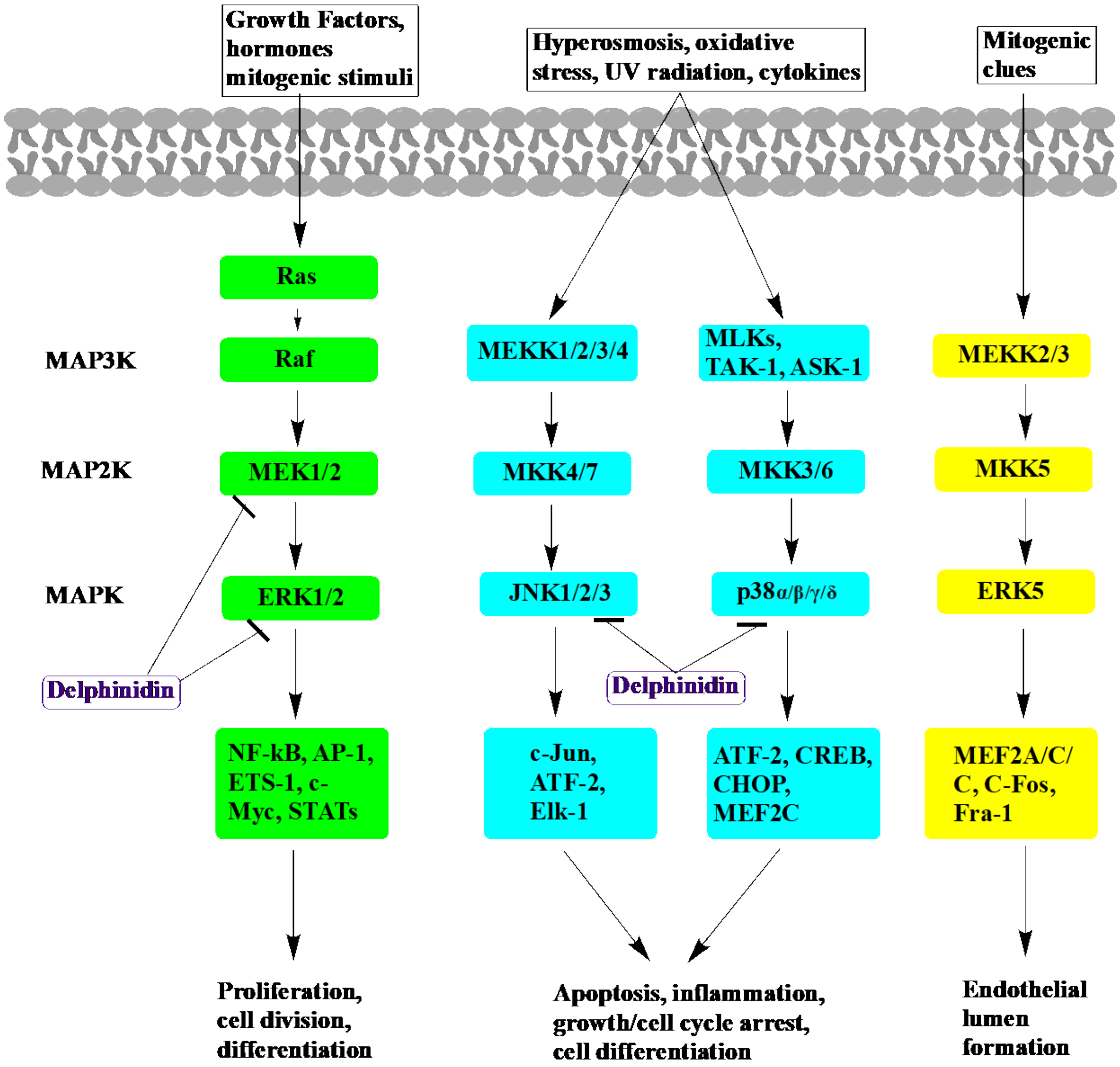

Figure S2. Schematic illustration of the effect of Delphinidin on the MAPK signaling pathway. Dp provokes apoptosis, hinder cell proliferation, division, differentiation and reduces inflammation by interfering with the regulation of proteins like MEK1/2, ERK1/2, p38 and JNK.

Table S1. Overview of some major patents granted for Delphinidin research.

Patent	Issue Date	Title	Summary	Inventor
**US5070212A**	December 3, 1991	**Intermediates useful for the synthesis of delphinidin chloride**	A process is provided for producing delphinidin (Dp)	Bruno Gabetta and Raffaello Giorgi
**KR100902768B1**	June 15, 2009	**Agents for preventing hair loss comprising delphinidin as an active ingredient**	The present invention relates to a composition comprising a Dp as an active ingredient.<br>It can improve collagenase activity inhibition, and skin conditions, such as excellent anti-wrinkle efficacy, and excellent hair growth promoting or hair loss prevention effect by the molecular mechanisms, such as the promotion of collagen synthesis (skin conditions).<br>Dp can be safely applied to the cosmetic, pharmaceutical and food compositions.	Lee and Jong Seong
**CN102796154B**	April 15, 2015	**Method for separating and preparing high-purity acylated delphinidin derivatives from eggplant peel**	The present invention relates to an isolated eggplant skin prepared from 3- [4-p- coumaroyl -L- rhamnopyranosyl - (1 → 2) -D- glucopyranoside] -5-D- glucopyranosyl.<br>The method is simple to operate, purity of glucopyranoside Dp was 99.79% or more, and may be implemented large-scale preparation.	Jing Dong, Ying Zhao, Shujuan Sun Xiangjun and Shen Shanqi
**CN103585292B**	December 9, 2015	**One application of delphinidin hair extract in the preparation of anticancer drug development**	The present invention relates to a new use of hair development Dp extract in the preparation of anticancer drugs.	Shang Xiyong and Zhang Xukun
**EP2854553B1**	December 21, 2016	**Use of delphinidin against *Staphylococcus aureus***	The invention involves the use of delphinidin or salts there of or of a complex comprising Dp for at least partially neutralizing an object contaminated with bacteria selected from the group consisting of antibiotics-resistant *Staphylococcus aureus* and antibiotics-sensitive *S. aureus*.	Nowbert Roewer and Jens Broscheit

**US9949947B2** US951104	April 24, 2018	**Delphinidin for combating melanoma cells**	The invention relates to a composition comprising: a complex of Dp and a sulfoalkyl ether-β-cyclodextrin and/or delphinidin and the salts thereof as a first component; and a tumor-necrosis factor related apoptosis-inducing ligand (TRAIL) as a second component, for use in the treatment of malignant melanoma.	Nowbert Roewer and Jens Broscheit
**US9925274B2**	27 March 2018	**Delphinidin complex as an antiphlogistic or immunosuppressive active ingredient**	The invention considers the use of complex comprising of Dp and sulfoalkyl ether β-cyclodextrin (enhances solubility and stability of delphinidin) as an antiphlogistic and immunosuppressive agent.	Norbert Roewer and Jens Broscheit
